# Combination of Antimicrobial Starters for Feed Fermentation: Influence on Piglet Feces Microbiota and Health and Growth Performance, Including Mycotoxin Biotransformation *in vivo*

**DOI:** 10.3389/fvets.2020.528990

**Published:** 2020-10-16

**Authors:** Laurynas Vadopalas, Modestas Ruzauskas, Vita Lele, Vytaute Starkute, Paulina Zavistanaviciute, Egle Zokaityte, Vadims Bartkevics, Iveta Pugajeva, Ingars Reinolds, Sarunas Badaras, Dovile Klupsaite, Erika Mozuriene, Agila Dauksiene, Romas Gruzauskas, Elena Bartkiene

**Affiliations:** ^1^Institute of Animal Rearing Technologies, Lithuanian University of Health Sciences, Kaunas, Lithuania; ^2^Microbiology and Virology Institute, Lithuanian University of Health Sciences, Kaunas, Lithuania; ^3^Department of Physiology and Anatomy, Lithuanian University of Health Sciences, Kaunas, Lithuania; ^4^Department of Food Safety and Quality, Lithuanian University of Health Sciences, Kaunas, Lithuania; ^5^Institute of Food Safety, Animal Health and Environment BIOR, Riga, Latvia; ^6^Department of Food Science and Technology, Kaunas University of Technology, Kaunas, Lithuania

**Keywords:** fermentation, feed, piglets, microbiota, blood parameters, growth performance, mycotoxins

## Abstract

The aim of this study was to apply a combination of the microbial starters *Lactobacillus uvarum* LUHS245, *Lactobacillus casei* LUHS210, *Pediococcus acidilactici* LUHS29, and *Pediococcus pentosaceus* LUHS183 for feed fermentation and to evaluate the influence of fermentation on feed acidity and microbiological characteristics, as well as on the piglet feces microbiota, health, and growth performance. Additionally, mycotoxin biotransformation was analyzed, including masked mycotoxins, in feed and piglet feces samples. The 36-day experiment was conducted using 25-day-old Large White/Norwegian Landrace (LW/NL) piglets with an initial body weight of 6.9–7.0 kg, which were randomly distributed into two groups (in each 100 piglets): control group, fed with basal diet (based on barley, wheat, potato protein, soybean protein concentrate, and whey powder), and treated group, fed with fermented feed at 500 g kg^−1^ of total feed. Compared to a commercially available lactic acid bacteria (LAB) combination, the novel LAB mixture effectively reduced feed pH (on average pH 3.65), produced a 2-fold higher content of L(+) lactic acid, increased viable LAB count [on average 8.8 log_10_ colony-forming units (CFU) g^−1^], and led to stable feed fermentation during the entire test period (36 days). Fecal microbiota analysis showed an increased number of probiotic bacteria in the treated group, particularly *Lactobacillus*, when compared with the control group at the end of experiment. This finding indicates that fermented feed can modify microbial profile change in the gut of pigs. In treated piglets' blood (at day 61), the serum high-density lipoprotein (HDL) cholesterol and triglycerides (TG) were significantly higher, but the levels of T4, glucose, K, alkaline phosphatase (AP), and urea were significantly decreased (*p* ≤ 0.05) compared with the control group. Mycotoxin analysis showed that alternariol monomethyl ether (AME) and altenuene were found in 61-day-old control piglets' feces and in fermented feed samples. However, AME was not found in treated piglets' feces. Feed fermentation with the novel LAB combination is a promising means to modulate piglets' microbiota, which is essential to improve nutrient absorption, growth performance, and health parameters. The new LAB composition suggests a novel dietary strategy to positively manipulate fermented feed chemicals and bio-safety and the piglet gut microbial ecology to reduce antimicrobials use in pig production and increase local feed stock uses and economical effectiveness of the process.

## Introduction

Veterinary drugs are widely used in animal production. This use has become a problem because pathogens develop resistance to antimicrobials, and the drugs can reach the soil and water through the animal excreta and act as serious environment pollutants (1–3). Research has suggested a myriad of nutritional strategies to improve animal health, productivity, and production quality. Most of these methods use feed supplements (plant and/or microbial) that stimulate a suitable intestinal ecosystem. Modification of intestinal microbiota is important for the health status of the pigs (4). Many lactic acid bacteria (LAB) are desirable intestinal microorganisms that can survive at surfaces of the gastrointestinal tract. Most of the LAB can ferment carbohydrates and reduce pH, an action that leads to a more acidic environment and suppression of pathogenic bacteria growth (5). Some LAB strains possess antimicrobial properties because they have the ability to produce substances [bacteriocins or bacteriocin-like inhibitory substances (BLIS)] that have the capacity to inhibit pathogens and that make them more specific anti-pathogenic agents (6). Our previous studies showed that *Lactobacillus uvarum* LUHS245, *Lactobacillus casei* LUHS210, *Pediococcus acidilactici* LUHS29, and *Pediococcus pentosaceus* LUHS183 strains inhibit a variety of pathogenic and opportunistic microorganisms *in vitro* (7). For instance, LUHS245 strain showed antimicrobial activity against methicillin-resistant *Staphylococcus aureus*, LUHS 210 and LUHS29 showed antimicrobial activity against *Salmonella enterica*, while hemp seed fermented with LUHS183 and LUHS245 showed inhibition of *Pasteurella multocida* (8).

There are published results about the effects of viable LAB on pigs' zootechnical parameters, intestinal microbiota, and gut health (9). Additionally, LAB strains have anti-infectious properties, e.g., reduction of *Salmonella* and enterotoxigenic *Escherichia coli* colonization (10). LAB-mediated fermentation can reduce toxins in feed and, during the fermentation, some of the microbial starters excrete enzymes that may transform mycotoxins into non-toxic compounds. However, fermentation can lead to the formation of masked mycotoxins, and special attention must be paid to control these processes. Additionally, LAB might lead to metabolic disorders in the host (11).

During the LAB metabolism, excreted lactic acid, in most of the cases, is a combination of the L-(+)- and D-(−) isomers. D(−) lactic acid cannot be metabolized by mammals; for this reason, it can cause acidosis, i.e., a disturbance in the acid–alkali balance (12). Therefore, in this study, we used LAB starters (previously tested *in vitro*) with antimicrobial properties against pathogenic and opportunistic strains. We examined whether this activity altered the piglet microbiota, an action that might improve the animals' health and productivity. In this study, we hypothesized that administration of a LAB combination with antimicrobial characteristics may reduce pathogenic and opportunistic strain concentration in intestine of piglets. Furthermore, modifying intestinal microbiota might improve piglets' health and growth performance.

The aim of this study was to apply a combination of the microbial starters *L. uvarum* LUHS245, *L. casei* LUHS210, *P. acidilactici* LUHS29, and *P. pentosaceus* LUHS183 for feed fermentation and to evaluate the influence of fermentation on feed acidity and microbiological characteristics, as well as on the piglet feces microbiota, health, and growth performance. Additionally, mycotoxin biotransformation was analyzed, including masked mycotoxins, in feed and piglet feces samples.

## Materials and Methods

**Figure 1:**
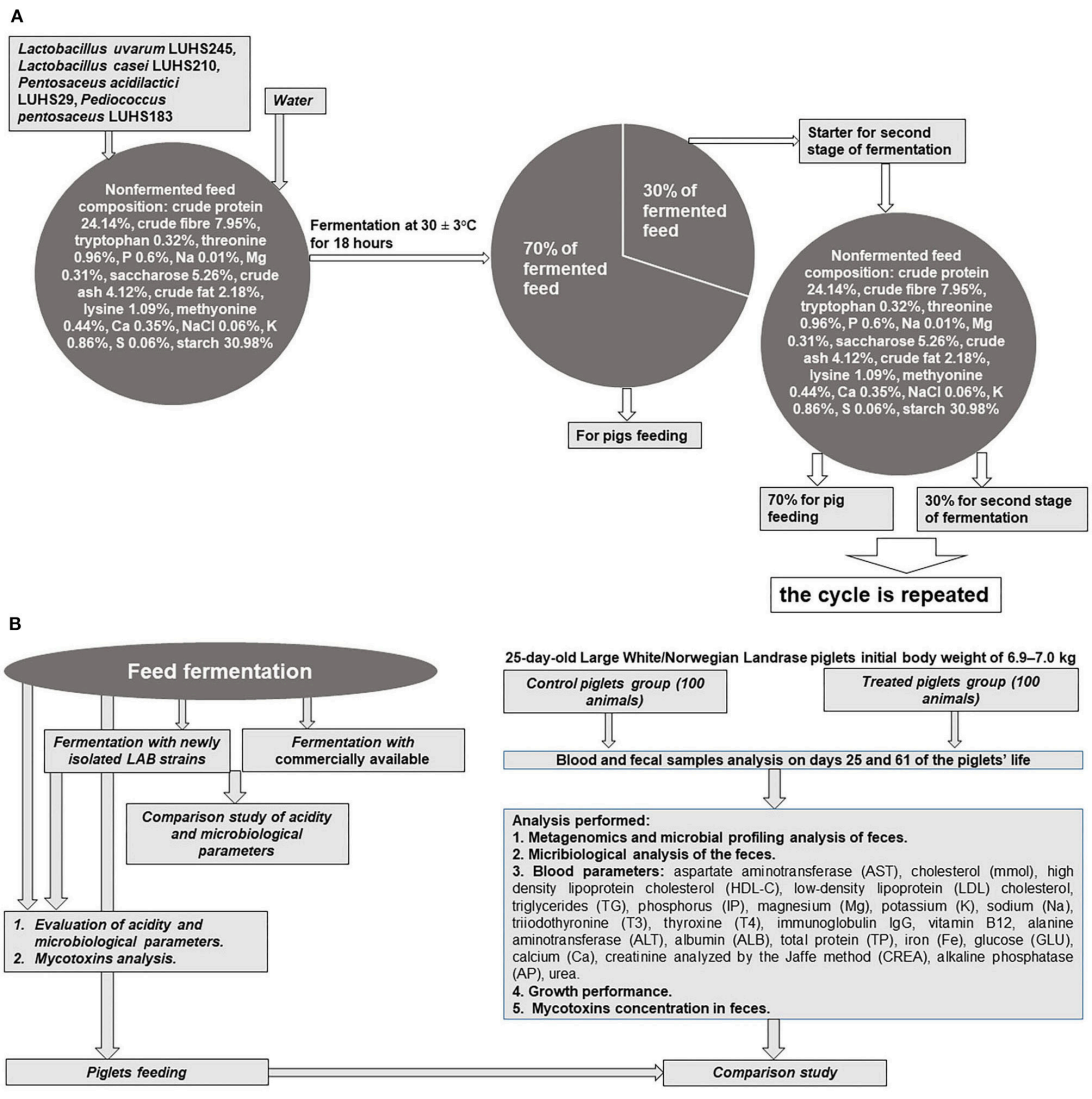
**(A,B)** Principal scheme of experiment.

The whole experiment principal scheme is shown in Figures 1A,B.

### Fermented Feed Preparation and Analysis

#### LAB Strains Used for Feed Fermentation

The *L. uvarum* LUHS245, *L. casei* LUHS210, *P. acidilactici* LUHS29, and *P. pentosaceus* LUHS183 strains were obtained from the Lithuanian University of Health Sciences collection (Kaunas, Lithuania). Our previous studies showed that the abovementioned strains inhibit various pathogenic and opportunistic microorganisms and are suitable for fermentation of various cereal substrates (5, 7, 13, 14). The abovementioned LAB strains were stored at −80°C in a Microbank system (Pro-Lab Diagnostics, UK) and separately propagated in de Man-Rogosa-Sharpe (MRS) broth (CM 0359, Oxoid Ltd., Hampshire, UK) at 30 ± 3°C for 48 h before their use for feed fermentation.

#### Feed Fermentation

The feed (composition: crude protein–19.00%, crude fiber–3.15%, crude oil and fats–6.51%, lysine–1.45%, methionine–0.55%, tryptophan–0.26%, threonine–0.93%, Ca–0.90%, total P–0.59%, and Na–0.20%), water, and LAB strain (equal parts of each strain by volume) suspension (3% from dry matter of feed mass, v/m), containing 8.9 log_10_ CFU ml^−1^, was fermented at 30 ± 2°C for 18 h. The final moisture content of the feed was 60 g 100 g^−1^. The moisture content was determined by drying the samples at 103 ± 2°C to a constant weight (15). Whole fermented feed mass (100%) was divided into two parts (30 and 70%, by mass): 70% of the fermented feed was used for piglet feeding, and 30% of fermented feed was used as a starter for additional feed fermentation cycles (Figure 1A). Non-fermented feed samples were analyzed as the control. In addition, a commercial LAB combination for feed fermentation was tested: *Lactobacillus plantarum* 1k2079, *P. pentosaceus* 1k2103, and *Lactococcus lactis* 1k2082 (H. Wilhelm Schaumann GmbH, Pinneberg, Germany). With the commercially available and newly developed LAB composition, fermented feed samples were analyzed every 18 h during the first 6 days to compare its pH and viable LAB counts (the main parameters of fermentation). Further, from the fifth day of fermentation, analyses were performed every 5 days.

#### Evaluation of Fermented Feed Acidity and Microbiological Parameters

The pH of samples was measured and recorded using a pH electrode (PP-15; Sartorius, Goettingen, Germany). Concentrations of L(+) and D(−) lactic acid isomers were determined with a specific Megazyme assay Kit (Megazyme Int., Bray, Ireland).

Evaluation of the LAB count was performed according the ISO 15214:1998 method (16), described in detail by Bartkiene et al. (13). The number of microorganisms was counted and expressed as log_10_ of colony-forming units per gram (CFU g^−1^). All results are expressed as the mean of three determinations.

### *In vivo* Experiment With Piglets

#### Animals and Housing

All animal procedures were conducted according to the EU Directive (17) of the European Parliament and of Council from 22 September 2010 on the protection of animals used for scientific purposes and Requirements for the Keeping, Maintenance, and Use of Animals Intended for Science and Education Purposes, approved by the order of the Lithuanian Director of the State Food and Veterinary Service (31/10/2012, No. B1-866) [(18); Figure 1B]. The study was conducted at a pig farm in the Klaipeda district (Kontvainiai, Lithuania) and at the Institute of Animal Rearing Technologies, Lithuanian University of Health Sciences (Kaunas, Lithuania). A 36-day experiment was conducted using 25-day-old Large White/Norwegian Landrace (LW/NL) piglets (100 piglets in each group) with an initial body weight of 6.9–7.0 kg. The weaner piglets were kept in a section with two climate zones. The first had a heated concrete floor (36°C) and roof on it, and the second had plastic piglet floors and optimum ventilated air and temperature for the active period. Drinking water and compound liquid feed were available *ad libitum* throughout the trial. Antibiotic treatment was not applied.

#### Experimental Design and Diets

The piglets were distributed into two groups (each of 100 animals), and samples (feces and blood) from 10 animals per group were collected. Two dietary treatments were compared: (i) non-fermented basal diet and (ii) fermented basal diet. Fermented feed comprised 500 g kg^−1^ of total feed; it was included in the diet of treated group beginning at day 25 of life until day 61. Evaluation of piglets' growth performance was performed by testing 100 piglets from each group. The basal feed was formulated according to the nutritional requirements prescribed in the Nutrient Requirements of Swine (19). The feed composition and nutritional value are shown in Table 1. Dietary contents were analyzed according to the AOAC recommendations (20).

**Table 1:** Diet composition.

	**Control group**	**Treated group**
**Ingredients (%)**		
Barley	38.40	33.25
Rapeseed meal	–	25.00
Wheat	32.12	25.02
Full fat soya-beans (extruded)	9.30	–
Potato protein	5.00	2.00
Soybean protein concentrate	2.00	–
Whey powder	5.80	5.80
Sunflower oil	2.72	4.51
Limestone	1.48	1.1
NaCl	0.38	0.35
Monocalcium phosphate	0.33	0.41
L-Lysine sulfate	0.87	1.1
DL-Methionine	0.25	0.16
*Acidal NC (formic and acetic acids)*	0.30	0.30
^a^Vitamins and trace elements *(premix)*	1.00	1.00
*Bredol 683*	0.05	0.00
**Nutritional value**		
ME swine (MJ/kg)	13.86	13.95
Crude protein (%)	19.00	19.00
Crude oil and fats (%)	6.51	6.51
Crude fiber (%)	3.15	5.14
Lysine (%)	1.45	1.45
Methionine (%)	0.55	0.55
Threonine (%)	0.93	0.94
Tryptophan (%)	0.26	0.25
Methionine + Cystine (%)	0.87	0.88
Ca (%)	0.90	0.90
Total P (%)	0.59	0.62
Available P (%)	0.37	0.38
Na (%)	0.20	0.21

ME, metabolizable energy.

a *Composition of premix per 1 kg of feed: Vitamin A–18,180 IU; vitamin D3–2040 IU; vitamin E–161 mg kg^−1^; vitamin K3–5.03 mg; thiamine–3.64 mg; riboflavin–9.16 mg; choline chloride–404 mg; pyridoxine–4.60 mg; vitamin B12–0.05 mg; niacin–41 mg; pantothenic acid–22.85 mg; folic acid–1.85 mg; biotin–0.21 mg; Fe–152 mg; Cu–101 mg; Zn–91 mg; Mn–80 mg; I–0.81 mg; Co–0.53 mg; Se–0.30 mg*.

#### Metagenomics and Microbial Profiling Analysis

Before the experiment, feces from 25-day-old control and treated piglets were collected from 10 piglets per group. The same procedure, using 10 piglets per group, was used at the end of the experiment (day 61 of the piglets' life) to generate representative samples of feces content from both animal groups. The DNA from each sample was kept in DNA/RNA Shield 1:10 (R1100-250, Zymo Research, USA) at −70°C before DNA extraction. DNA was extracted with a fecal DNA MiniPrep kit (D6010, Zymo Research, USA). Library preparation, metagenomic sequencing, and taxonomic characterization of reads was performed as described previously (21). ZymoBIOMICS Microbial Community Standard (D6300, Zymo Research, USA) was used as a microbiome profiling quality control. The results of taxonomic classification were visualized using the interactive online platform (https://genome-explorer.com).

#### Microbiological Analysis of Fecal Samples

The piglets' fecal samples were collected before and after the experiment, stored in vials (+4°C) with a transport medium (Fecal Enteric Plus, Oxoid, Basingstoke, UK), and analyzed on the same day. MRS agar was used to determine the LAB count in the feces. Violet Red Bile Glucose (VRBG) agar (Oxoid Ltd., Basingstoke, United Kingdom) was used to determine the total count of enterobacteria (TCE). Plate Count Agar (Biolife Italiana Srl, Milan, Italy) was used to determine the total aerobic and facultative anaerobic bacteria count (TCM) in the feces. The results are expressed as a log_10_ of CFU g^−1^ of a sample.

#### Blood Analysis

Piglets were bled (10 animals from each group) from the jugular vein into vacuum blood tubes (BD Vacutiner, United Kingdom) before the morning feeding. Tubes with clot activator were used for biochemical examination. Blood biochemical variables were evaluated before and after the experiment (on days 25 and 61 of the piglets' life). The following parameters were included: aspartate aminotransferase (AST), cholesterol (mmol), high-density lipoprotein cholesterol (HDL-C), low-density lipoprotein (LDL) cholesterol, triglycerides (TG), phosphorus (IP), magnesium (Mg), potassium (K), sodium (Na), triiodothyronine (T3), thyroxine (T4), immunoglobulin IgG, vitamin B12, alanine aminotransferase (ALT), albumin (ALB), total protein (TP), iron (Fe), glucose (GLU), calcium (Ca), creatinine analyzed by the Jaffe method (CREA), alkaline phosphatase (AP), and urea. They were analyzed with an automatic biochemistry analyzer “SIEMENS ADVIA 1800” (Siemens Healthcare GmbH, Germany) and immunochemical analyses [triiodothyronine (T3) and thyroxine (T4)] by analyzer “SIEMENS ADVIA Centaur XP” (Siemens Healthcare GmbH, Germany) in the accredited laboratory “Anteja” (Klaipeda, Lithuania).

#### Evaluation of Piglets' Growth Performance

Group body weight (BW) was recorded on days 25, 32, 39, 46, 53, and 61 of age using an electronic weighing system (model type: IT1000, SysTec GmbH Bergheim, Germany). The feed efficiency (FE) was determined from feed intake and BW, which was recorded on the same days as BW using a WEDA (Dammann & Westerkamp GmbH, Germany) automated feeding system that has an electronic flowmeter and weighing system.

#### High-Performance Liquid Chromatography Coupled to Time of Flight High-Resolution Mass Spectrometry (HPLC-TOF-HRMS) for Mycotoxin Analysis

The standards of beauvericin (BEA, ≥95%), enniatin A (ENN A, ≥99%), enniatin A1 (ENN A1, ≥99%), enniatin B (ENN B, ≥99%), enniatin B1 (ENN B1, ≥99%), meleagrin (MEL, ≥98%), cytochalasin A (CCA, ≥98%), cytochalasin B (CCB, ≥98%), cytochalasin C (CCC, ≥99%), cytochalasin D (CCD, ≥95%), cytochalasin E (CCE, ≥98%), cytochalasin J (CCJ, ≥95%), cytochalasin H (CCH, ≥95%), 15-acetyldeoxynivalenol (15-AcDON, ≥99%), 3-acetyldeoxynivalenol (3-AcDON, ≥99%), tentoxin (TNX, ≥99%), citreoviridin (CVD, ≥95%), stachybotrylactam (SBL, ≥95%), alternariol monomethyl ether (AME, ≥98%), dihydrochalasin B (DTC B, ≥98%), and fusaric acid (FA, ≥98%) were purchased from Cayman Chemical (Ann Arbor, MI, USA). Deoxynivalenol (DON, 98.3%), aflatoxins (AFB1, AFB2, AFG1, AFG2, ≥99%), aflatoxin M1, aflatoxin Ro (aflatoxicol), HT-2 toxin (HT-2, 99%), T-2 toxin (T-2, 99%), sterigmatocystin (STC, 99.7%), zearalenone (ZEN, 99,66%), ochratoxin A (OTA, 99%), fumonisins B1 and B2 (FB1, 98%; FB2, 97.5%), fusarenon-X (FUS-X, 99.4%), and deoxynivalenol-3-glucoside (D3G, 96%) were acquired from Romer Labs (Tulln, Austria). Neosolaniol (NEO, 99%), anisomycin (ANC, 98.9%), T-2 toxin tetraol (T-2TET, >99%), apicidin (API, >99%), ansamitocin P3 (AN P3, 99%), altenuene (ALT, 99.3%), alternariol (AOH, >98%), cerulenin (CER, 98%), chaetocin (CTC, 99%), 15-acetoxyscirpenol (15-AcS, >98%), T-2 toxin triol (T-2TRI, 99%), fumonisin B3 (FB3, 99%), myriocin (MYR, 99%), brefeldin A (BRF A, 99.9%), 17-dimethylaminoethylamino-17-demethoxygeldanamycin (17-DMAG, 99.4%), altertoxin I (ATX I, 99%), 17-(allylamino)-17-demetoxygeldanamycin (17-AAG, 99%), aflatoxicol (AFL, 99%), chaetoglobosin A (CHG A, 99%), verruculogen (VCL, >99%), wortmannin (WTM, 99%), helvolic acid (HA, 99%), ochratoxin B (OTB, 99%), destruxin A (DTX A, 99%), destruxin B (DTX B, 99%), paxilline (PXL, 99%), penitrem A (PN A, >99%), gliotoxin (GTX, >99%), curvularin (CVL, 99%), bafilomycin A1 (BFA1, >99%), and bafilomycin B1 (BFB1, >99%) were purchased from Fermentek (Jerusalem, Israel), while mycophenolic acid (MPA, >99%), penicillic acid (PA, >99%), and roquefortine-C (ROQ-C, >99%) were purchased from Santa Cruz Biotechnology (Dallas, TX, USA).

Standard stock solutions of all mycotoxins were prepared in acetonitrile, methanol, or their mixtures with DMSO, with the exception of BEA and enniatins that were kept in DMF. The spiking solutions and calibration standards were prepared by serial dilution of stock solutions and were stored in UV-protected glassware at 4°C (22).

The samples were prepared using a modified QuEChERS method. HPLC-TOF-HRMS analysis was performed on an UltiMate 3000 (Thermo Fisher Scientific, USA) HPLC system coupled to a Compact Q-ToF time-of-flight mass spectrometer (Bruker, Germany). Chromatographic separation was performed on a reversed-phase analytical column (Kinetex C_18_, 1.7 μm, 100 Å, 50 × 3.00 mm; Phenomenex, USA) at a 0.35 ml min^−1^ flow rate. The analysis was performed in positive full scan mode for all mycotoxins over the *m/z* scanning range from 50 to 1,000. The mass extraction window applied for quantification purposes was set to ±5 ppm at 10,000 full with at half maximum (FWHM) resolution. Data acquisition was controlled by HyStar 3.2. software (Bruker Daltonik GmbH, Bremen, Germany), and data analysis was performed with QuantAnalysis 4.3. software (Bruker Daltonik GmbH, Bremen, Germany).

### Statistical Analysis

In order to evaluate the influence of fermentation on feed characteristics, data were subjected to analysis of variance (ANOVA) and paired *t*-test column statistics. Comparisons were considered significant when *p* ≤ 0.05. All feed sample analytical experiments were performed in triplicate (*n* = 3). ANOVA was also performed to assess the effects of treatment with fermented feed on piglet' parameters. When the ANOVA indicated a significant treatment effect, the means were separated using Duncan's multiple range tests. In the tables, piglets' sample results are presented as mean values with pooled standard errors (*n* = 10).

## Results and Discussion

### Characteristics of Fermented Feed

#### pH and LAB Count for Feed Fermented With Commercial and Novel LAB Combinations

**Figure 2:**
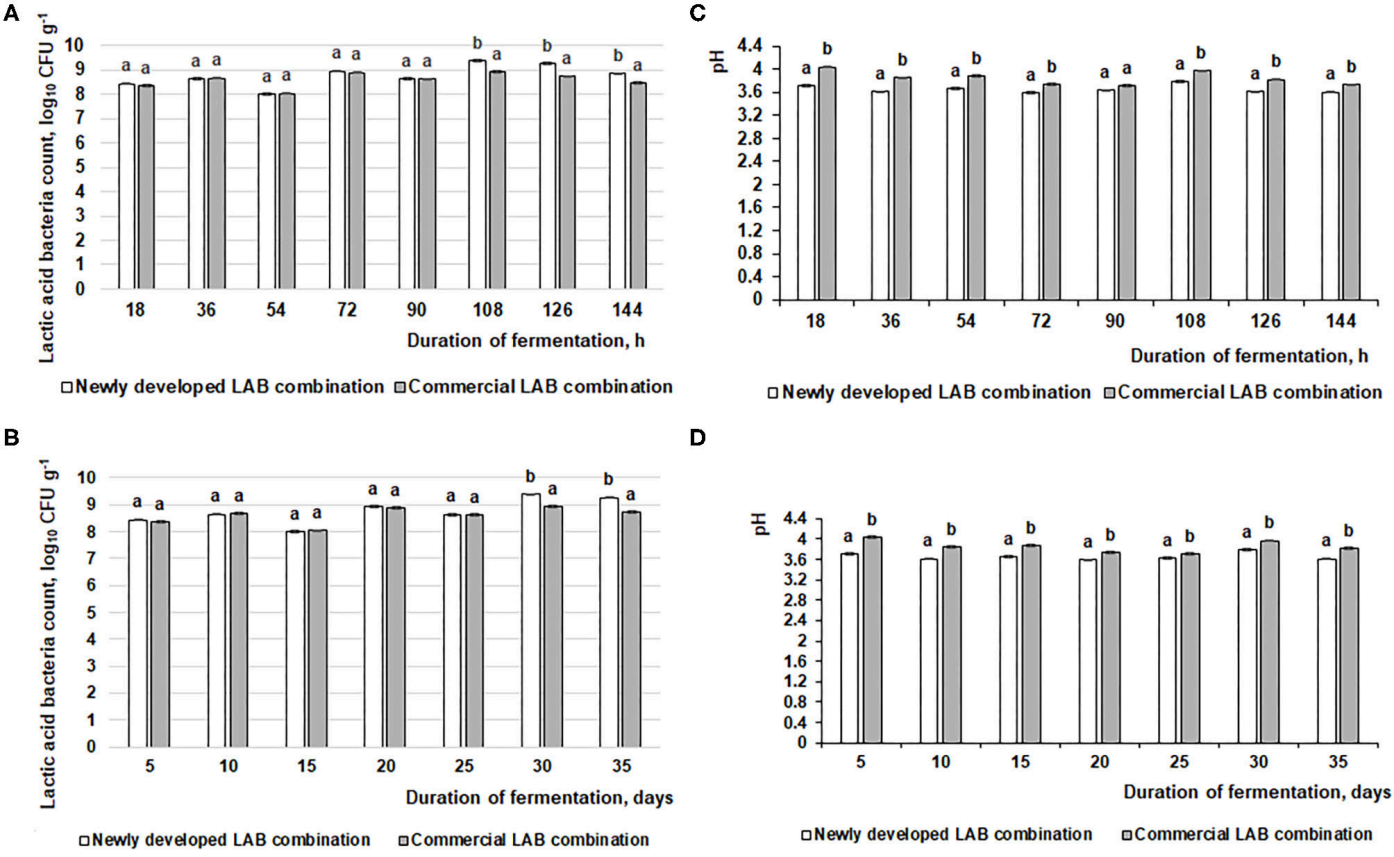
Changes in pH and lactic acid bacteria (LAB) count (log_10_ CFU g^−1^) during the feed fermentation. **(A,C)** LAB count and pH of the feed after 18, 36, 54, 72, 90, 108, 126, and 144 h. **(B,D)** LAB count and pH of the feed after 5, 10, 15, 20, 25, 30, and 35 days. Data are expressed at the mean ± standard deviation (*n* = 10). Data were statistically compared with a paired *t*-test and column statistics; *p* ≤ 0.05 was considered significant. Each parameter, means followed by different letters are significantly different (*p* ≤ 0.05).

The changes in fermented feed pH and LAB count during the fermentation with the commercial or novel LAB combinations are presented in Figure 2. The feed fermentation process was performed according to the scheme in Figure 1A, and pure LAB cultures were not added during the experimental period. From 18 to 144 h of fermentation, in most of the cases (except samples after 90-h fermentation), the newly developed LAB combination significantly reduced the feed pH compared to the commercial combination (average pH was 3.65 and 3.84, respectively; Figure 2C). This same tendency was noted during the entire 35-day experimental period (35 days). On average, the pH for samples fermented with the commercial LAB combination was 3.86, while it was 3.66 with the newly developed LAB combination (Figure 2D).

When comparing the viable LAB count in fermented feed samples, after 18, 36, 54, 72, and 90 h of fermentation, there were significant differences between the LAB counts in feed samples fermented with the commercial or newly developed LAB combination. On average, the LAB count in fermented feed was 8.5 log_10_ CFU g^−1^ (Figure 2A). After 108, 126, and 144 h, the LAB count was significantly higher in feed samples fermented with the newly developed compared to the commercial LAB combination (on average, 9.2 log_10_ CFU g^−1^ and 8.7 log_10_ CFU g^−1^, respectively). There was a similar tendency for this measure during the entire 35-day evaluated period: 8.6 log_10_ CFU g^−1^ in feed fermented with the commercial LAB combination and 8.8 log_10_ CFU g^−1^ for feed fermented with the novel combination (Figure 2B).

LAB are popular microorganisms used for fermented liquid and solid-state feed preparations to reduce the pH of fermentable substrates by converting carbohydrates to organic acids (23). In fermented feed, the inhibition of pathogenic and opportunistic strains is explained by evaluating the number of Enterobacteriaceae and molds. High content of LAB with anti-pathogenic characteristics may have a positive influence on the microbial population in the intestine. It was published that *Lactobacillus brevis, Lactobacillus acidophilus, Lactobacillus reuteri*, and *L. plantarum*, can reduce *E. coli* in the intestine of weaned piglets (24). LAB are natural inhabitants of the intestine that can survive in gastrointestinal tract and perform nutritional compounds' degradation activity. Their possibility to adherence to gastrointestinal tract surfaces may reduce colonization of pathogens (25). In feed fermentations, the main metabolite of LAB is lactic acid, the concentration of which should be above 150 mmol L^−1^ to inhibit endogenous pathogens in the fermentable substrate (26). However, lactic and acetic acid—and the ethanol concentrations—should be controlled in fermented feed to avoid causing undesirable palatability of end products and/or acidosis (27). Furthermore, a decline in pH may partially unbalance the secretion of hydrochloric acid in the stomach of young piglets. It can reduce the stomach's ability to digest and absorb feed and kill off pathogens (28). Fermented feed pH, one from the most important fermented feed quality indicators, allows experimenters to evaluate the nutritional value and biosafety of the end product to suppress pathogenic bacteria (29). pH is a very important fermentation indicator and should be monitored to control the fermented feed preparation process. Optimal fermentation conditions occur when the fermentable substrate pH is 4.0–5.0; such a pH does not indicate overfermentation or uncontrolled fermentation (23, 30). Finally, desirable fermented feed characteristics are predominantly live probiotics, desirable technological microorganism metabolites and prebiotics, low counts of endogenous pathogens, and good sensory properties (23) According to this study, the abovementioned fermented feed characteristics were obtained by using the scheme shown in Figure 1, in which pure starter cultures are used in only the initial stage of the process.

#### L(+) and D(−) Lactic Acid Isomers Concentration in Fermented Feed

**Figure 3:**
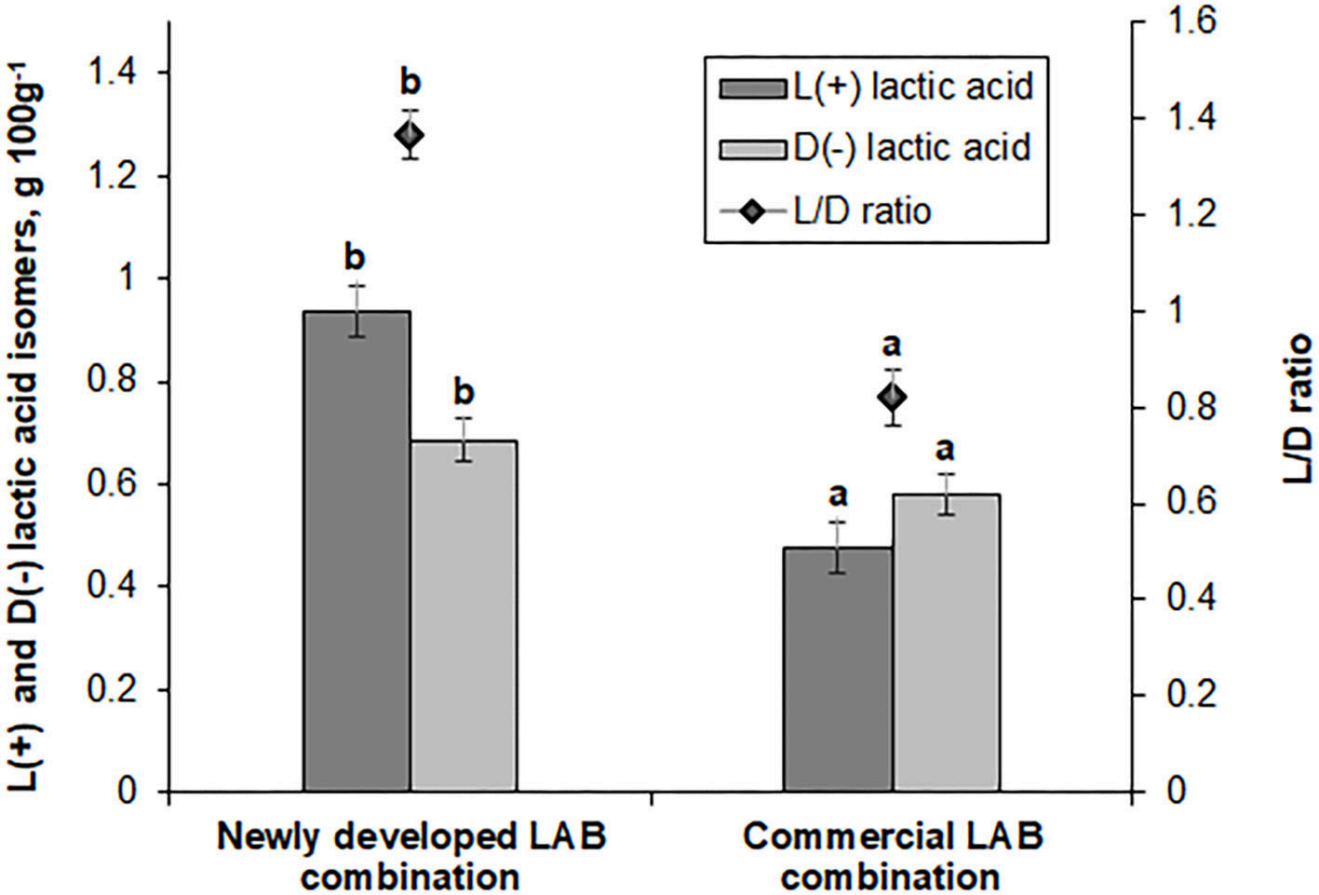
L(+) and D(−) lactic acid isomer concentrations (g 100 g^−1^) and ratio (L/D) in feed samples fermented with commercial or newly developed lactic acid bacteria (LAB) combinations. Data are expressed as the mean ± standard deviation (*n* = 10). Data were statistically compared with the paired *t*-test and column statistics. For each parameter, means followed by different letters are significantly different (*p* ≤ 0.05).

L(+) and D(−) lactic acid isomer concentration and the L/D ratio in fermented feed samples are shown in Figure 3. Feed samples fermented with the novel LAB combination exhibited a 2-fold higher L(+) lactic acid isomer concentration and a 1.36-fold higher L/D ratio (compared with the feed samples fermented with the commercial LAB combination). Lactate is a major end product of LAB; however, increased lactic acid concentrations are often established in the feces of mammals that show some diseases. In farm animals, lactic acidosis is caused by an imbalance in lactic acid concentration (31, 32). This phenomenon increases mortality and reduces survival in neonatal pigs and humans. Therefore, lactate metabolism plays an important role in maintaining the host animal's health. Lactic acid can be metabolized *in vitro* into acetate and propionate in pig cecal digesta (33). L-lactic acidosis is the most common cause of metabolic acidosis in the critical care factor. It has been associated with a significant increase in mortality. L-lactic acidosis is defined by a blood L-lactate level of >5 mmol L^−1^ (34). D-lactic acid is the geometric isomer of L-lactate (a body metabolite). It is a metabolic end product of the intestinal flora (35). D-lactic acid is widely distributed within a body, and the concentration of D isomer is correlated with various diseases. Increases of D-lactic acid concentration can be influenced by short bowel syndrome, ischemia, and bacterial infection. D-lactic acid is potentially a feed quality indicator, which may indicate contamination with bacteria that causes undesirable changes in quality and taste (36).

### Influence of Fermented Feed on Pigs' Parameters

#### Microbial Profiles of Pig Feces

From 41,000 to 61,000 metagenomic sequence reads were obtained and analyzed (depending on the sample and period of investigation). Before the experiment, the most prevalent genus in both piglet groups was *Prevotella*: 33% and 25% from all of the genera in the control and experimental groups, respectively. The other most prevalent genera included *Barnesiella, Alloprevotella, Bacteroides, Clostridium, Escherichia*, and *Lactobacillus*. Such data are consistent with the findings from other authors who investigated microbiota in young piglets (2, 37–40). Overall, 179 and 151 genera with a prevalence ≥0.01% were detected in the fecal DNA of control and experimental animals, respectively (Supplementary Files 1, 2).

**Figure 4:**
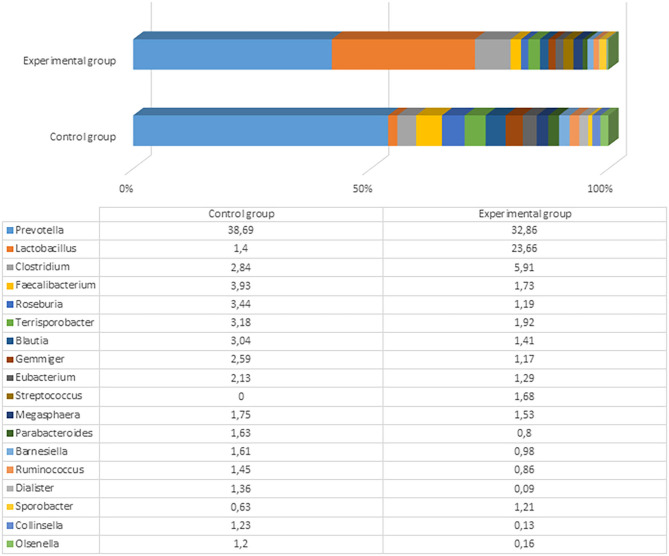
The most prevalent microbiota at a genus level (the prevalence at least 1% from all bacteria in control or either experimental group) in the fecal content of pig feces after the experiment (61st day).

After the experiment (day 61), there were significant differences among microbial profiles between the groups, although the number and variety of genera remained very similar (153 and 152 genera with a prevalence ≥0.01% in the control and experimental groups, respectively). The most prevalent genus in both groups was still *Prevotella*, but the prevalence of *Lactobacillus* was 6-fold higher in the experimental compared to control animals (23.7 vs. 3.9%). The differences of microbial profiles between the groups are presented in Figure 4 and Supplementary Files 3, 4.

The variety of the most prevalent bacteria (>1%) at the genus level was not very high: Only *Prevotella, Lactobacillus, Clostridium, Faecalibacterium, Roseburia, Terrisporobacter, Blautia*, and some other genera reached this level. Besides the higher *Lactobacillus* prevalence in the experimental pigs, there were higher numbers of *Clostridium* and *Streptococcus* in experimental pigs' fecal content compared with the control group. Among *Clostridium*, more than 60 species were detected in the experimental group, with the highest prevalence being of *Clostridium cellulovorans* (4.08%), *Clostridium celatum* (0.38%), and *Clostridium quinii* (0.31%). C. cellulovorans is a mesophilic and anaerobic cellulolytic bacterium that utilizes cellulose and hemicelluloses composed of xylose, fructose, galactose, and mannose (41). However, there is no clear information regarding functions of this species in the pig gut. Seven *Streptococcus* species were detected in the experimental group of pigs, including *Streptococcus lutetiensis, Streptococcus gallolyticus, Streptococcus danielleae, Streptococcus equinus, Streptococcus macedonicus, Streptococcus porcorum*, and *Streptococcus equi*. No streptococci were detected in the control animals at the end of the experiment. The fecal content in the control group contained higher amounts of *Faecalibacterium, Roseburia, Ruminococcus, Terrisporobacter, Blautia*, and some other microorganisms, although those differences were not very large between the groups. These distinctions were probably associated with high content of *Lactobacillus* in the experimental group that rendered a lower relative (percent) content of other bacterial genera. Clostridia from the genera *Roseburia, Blautia*, and *Ruminococcus* can help prevent pathogen colonization of the pig gastrointestinal tract by pathogens (42). Therefore, the microbial composition in the control group of pigs was also appropriate.

**Figure 5:**
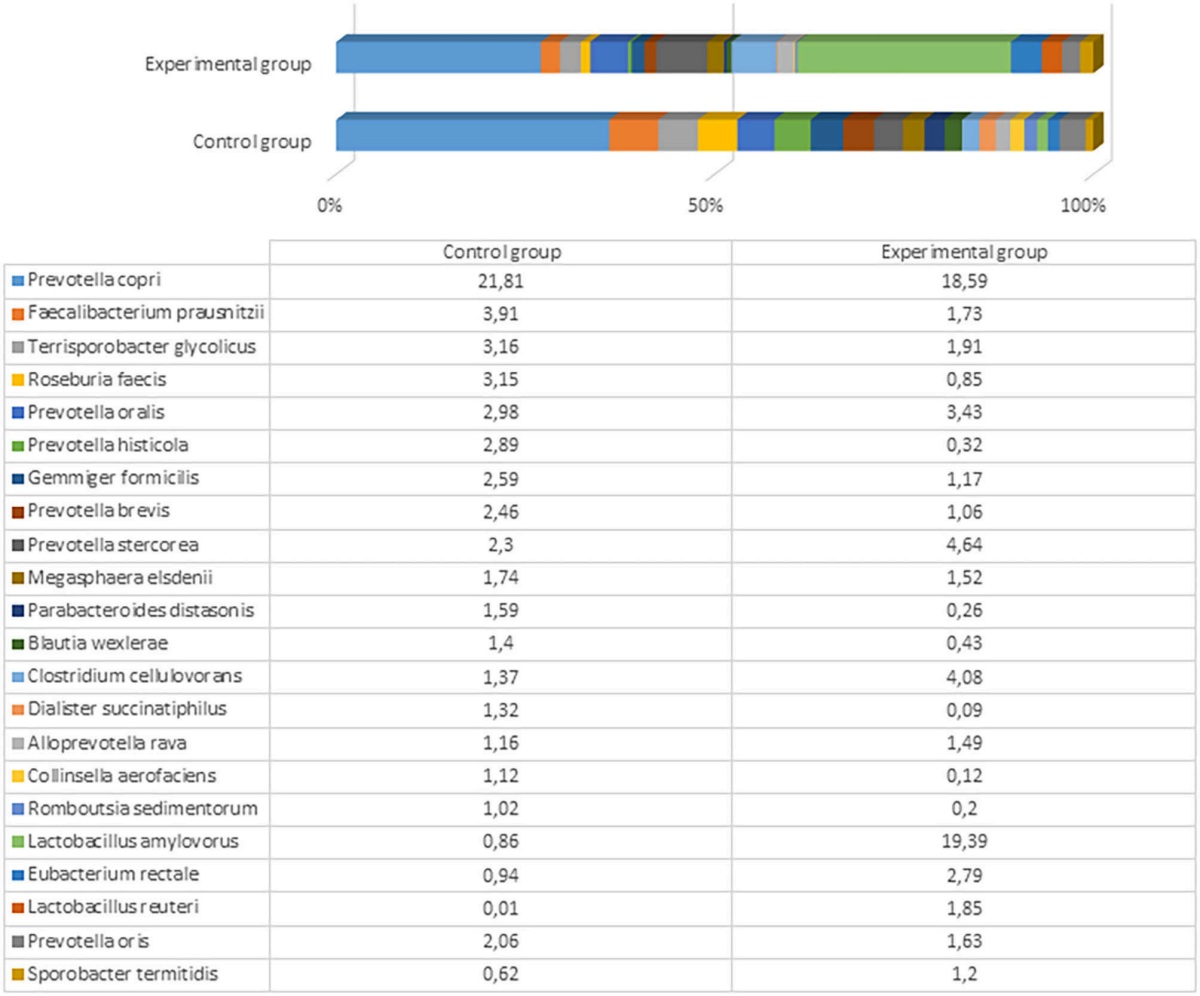
The most prevalent bacterial species (the prevalence at least 1% from all bacteria in control or either experimental group) in the fecal content of pig feces after the experiment (61st day).

The most prevalent bacterial species in the control pigs was *Prevotella copri* (21.81%), followed by *Faecalibacterium prausnitzii* (3.91%). Comparatively, the experimental group was predominated by *Lactobacillus amylovorus* (19.39%), followed by different *Prevotella* spp., including *P. copri* (18.59%), *Prevotella stercorea* (4.64%), and *Prevotella oralis* (3.43%). The bacterial species variety and difference between the groups are presented in Figure 5 and Supplementary Files 5, 6.

L. amylovorus is present in the intestines of piglets and exhibits several potential probiotic properties, including antimicrobial activity against enteric pathogens, in both *in vitro* and *in vivo* assays (43). We detected a marked difference in the amount of this bacterium in pigs fed with fermented and conventional feed. Although other microbiota detected in both experimental and control groups can be treated as normal or even probiotic, there were large differences in *Lactobacillus* that are known and recognized as beneficial and crucial microorganisms (i.e., to ensure good health) in the gut of pigs fed with treated food. Dietary changes can greatly shape the structure and function of gut microbiota. Various fermented feeds have been reported to exert beneficial effects on the pig microbiota during different growth states. The most common change is an increase in the LAB concentration, particularly in the stomach and small intestine (44). Moran et al. (45) reported that the ratio of LAB to coliform bacteria in the lower gut of pigs weaned using fermented liquid feed is shifted in favor of LAB. In contrast, this ratio is shifted in favor of the coliforms in piglets fed with dried feed. Another significant change in the microbial population of the gastrointestinal tract after feeding with fermented feed is an increase in the number of yeast cells (23). Yeast can bind enterobacteria surfaces and thereby block the binding of these bacteria to the gut epithelium (46). There are also opposite findings, where the fermented feed tends to decrease the population of LAB and anaerobic bacteria in general, especially in the large intestine, and increase the pH of the lower gut (26). Urlings et al. (47) hypothesized that fewer nutrients, including vitamins and amino acids, reach the large intestine, and this deficit promotes less microbial development and an increase in pH in the lower part of the gastrointestinal tract. A decrease in feed intake and body weight gain reportedly occurs due to impaired palatability after fermentation (44). The differences in those findings compared to this study might be associated with different microorganisms used for feed fermentation, the technological process, and distinct microbiome studies, as only recent achievements in molecular biology have allowed researchers to explore the microbiome more deeply, particularly regarding probiotic anaerobic bacteria. Previous studies were based on culturable methods; therefore, multiple species, including unculturable bacteria, were probably underestimated.

#### Influence of Fermented Feed on LAB, Total Enterobacteria Count (TEC), and Mold/Yeast M/Y Count in Piglets' Feces

The influence of fermented feed on TEC, LAB, and M/Y count in piglets' feces is shown in Table 2. TEC in the control and treated group feces decreased from day 25 to 61 (by 35.5 and 43.2%, respectively). However, TEC was significantly lower in the treated compared to the control group at the end of the experiment. There were no significant differences in the LAB count between groups; the average content was 7.2 log_10_ CFU g^−1^. The M/Y count was significantly higher (18.4%) at the beginning of the experiment in the treated compared with the control group. The same tendency occurred at the end of the experiment (M/Y was 9.6% higher compared with the control group). However, the difference between the M/Y count at the end of experiment was 1.9 times lower compared with the counts at the beginning of experiment. Demecková et al. (48) reported a lower number of coliforms and higher LAB count in the feces of piglets from sows fed with fermented liquid feed compared to piglets from sows fed with non-fermented or dry feed. In another study, there were fewer coliform bacteria in pigs that received a fermented feed diet, while differences in the number of LAB in the small intestine of differently fed pigs were not significant (44). A low pH, high lactic acid concentration, and high numbers of LAB in fermented feed are believed to be responsible for the decrease of enteropathogens (49). The fermented feed decreases pH and stimulates proteolytic activity in stomach, which is an important barrier against pathogens. These phenomena reduce the growth of undesirable pathogenic bacteria in the lower small intestine, cecum, and colon (50). Low pH plays a vital role in the inhibition of *Enterobacteriaceae* (51). In addition, it is important to use starter cultures that possess antimicrobial activity against gram-negative microorganisms (52). Our results are consistent with Väkeväinen et al. (53), who determined that both *L. lactis* A1MS3 and *P. pentosaceus* S0l10 possess antimicrobial properties. Starter cultures noticeably decrease *Enterobacteriaceae*, leading to a microbiologically safer end product. The pH decrease and increase of LAB and yeasts counts during the fermentation were in accordance with previous findings (54). At the end of the experiment, our results were similar with Nowak et al. (55), who reported that levels of yeast and molds in the cecal digesta are reduced. LAB are considered to be beneficial intestinal bacteria, whereas coliforms and *Salmonella* are considered to be major bacteria that often cause gut health problems such as diarrhea, especially in younger animals. Our results are consistent with Upadhaya et al. (56), who demonstrated that the LAB population is weakly influenced by fermented feed but the coliform population is significantly reduced. These finding indicate that gut microbiota is positively influenced by feed fermentation.

**Table 2:** Microbiological parameters [total enterobacteria count (TEC), lactic acid bacteria (LAB), and mold/yeast (M/Y) ratio] for feces from 25- and 61-day-old pigs.

**Microbiological parameters (log_**10**_ CFU g^**−1**^)**	**Pig groups**				***p***			
	**C25d**	**C61d**	**T25d**	**T61d**	**C25d × T25d**	**C25d × C61d**	**T25d × T61d**	**C61d × T61d**
TEC	7.6 ± 0.3	4.9 ± 0.2	8.1 ± 0.3	4.6 ± 0.2	0.011	0.0001	0.0001	0.0001
LAB	7.1 ± 0.3	7.2 ± 0.3	7.2 ± 0.1	7.3 ± 0.1	0.225	0.300	1.0	0.478
M/Y	4.9 ± 0.1	5.2 ± 0.2	5.8 ± 0.3	5.7 ± 0.2	0.016	0.035	0.194	0.0001

CFU, colony-forming units.

C, control group, fed with the basal diet; T, treated group, fed with the fermented feed; 25 d, 25-day-old piglets; 61 d–61-day-old piglets.

Data are expressed as the mean ± standard deviation (n = 10).

*Data were statistically compared with a paired t-test and column statistics; p ≤ 0.05 was considered significant*.

#### Piglet Blood Parameters

Piglet blood parameters are shown in Table 3. There were significantly higher serum ALB, T4, and Fe concentrations, as well as lower serum hepatic enzyme AST activity and LDL cholesterol, K, Ca, vitamin B12, and urea concentrations in the treated piglet blood samples before the feeding experiment. At day 61, serum HDL cholesterol and TG were significantly higher, and T4, glucose, K, AP, and urea were decreased (*p* ≤ 0.05) in blood from piglets fed with fermented compared with the control feed. Our study is consistent with Dong et al. (57), who reported that fermented feed improves the hematological profile and serum concentrations of total protein, albumin, and globulin and reduces serum triglyceride and cholesterol in weaned piglets. Increased serum glucose level in pigs supplemented with fermented feed was reported (58). The plasma protein concentration shows factors affecting the state of health: the hormone balance, nutritional status, water balance, etc. (59). The positive effect of *P. acidilactici* FT28 was consistent with a decreased serum TG concentration. Joysowal et al. (60) also observed a lower serum TG level by supplementing species-specific *P. acidilactici* and *L. acidophilus* in grower-finisher pigs. Blood analyses showed that the fermented feed increased glucose and decreased urea concentrations, data that are indicative of alterations in metabolism associated with the diet (61). Our findings are in agreement with Tretola et al. (61), namely, that these changes are due to the higher digestibility of the starchy feed and their higher glycemic index. Additionally, glucose—irrespective of insulin levels—decreases hepatic amino nitrogen conversion, an action that reduces the plasma nitrogen urea concentration (62).

**Table 3:** Blood parameters of the piglets.

**Blood parameters**	**C25d**		**T25d**		**C61d**		**T61d**		***p***			
	**AV**	***SD***	**AV**	***SD***	**AV**	***SD***	**AV**	***SD***	**C25d × T25d**	**C25d × C61d**	**T25d × T61d**	**C61d × T61d**
Aspartate aminotransferase (AST), U L^−1^	57.00	8.28	48.4	8.6	48.2	9.4	61.0	23.9	**0.0001**	**0.006**	0.290	0.265
Alanine aminotransferase (ALT), U L^−1^	42.2	7.2	43.8	7.9	76.4	17.7	89.4	42.9	0.057	**0.030**	0.153	0.466
Cholesterol (Chol), mmol L^−1^	1.71	0.15	1.65	0.24	2.58	0.32	2.60	0.21	0.299	**0.012**	**0.0001**	0.816
High-density lipoprotein cholesterol (HDL-Chol), mmol L^−1^	0.668	0.040	0.762	0.101	0.878	0.066	1.05	0.07	0.117	**0.005**	**0.003**	**0.0001**
Low density lipoprotein cholesterol (LDL-Chol), mmol L^−1^	0.808	0.154	0.676	0.146	1.47	0.21	1.24	0.09	**0.001**	**0.002**	**0.002**	0.065
Triglycerides (TG), mmol L^−1^	0.522	0.099	0.454	0.135	0.520	0.199	0.690	0.183	0.081	0.976	**0.013**	**0.003**
Total protein (TP), g L^−1^	48.3	2.9	50.7	12.4	54.3	5.3	54.7	2.0	0.706	0.048	0.575	0.836
Albumin (ALB), g L^−1^	33.2	3.8	46.6	4.4	33.8	5.3	33.2	2.6	**0.001**	0.547	**0.006**	0.735
Immunoglobulin IgG, g L^−1^	2.22	0.21	2.80	0.81	3.65	0.32	3.77	1.03	0.239	**0.002**	**0.016**	0.793
Triiodothyronine (T3), nmol L^−1^	1.50	0.32	1.28	0.46	1.53	0.21	1.52	0.14	0.125	0.642	0.333	0.731
Thyroxine (T4), μ dl^−1^	3.22	0.70	4.58	0.84	4.16	0.36	3.02	0.63	**0.003**	**0.042**	**0.006**	**0.018**
Glucose (GLU), nmol L^−1^	5.28	1.54	5.4	0.9	5.84	0.68	5.46	0.70	0.747	0.376	0.740	**0.001**
Phosphorus (IP), mmol L^−1^	3.06	0.26	2.95	0.33	3.66	0.13	3.77	0.41	0.090	**0.016**	**0.003**	0.561
Magnesium (Mg), mmol L^−1^	0.924	0.129	0.968	0.079	1.12	0.14	1.12	0.15	0.265	**0.002**	0.072	0.783
Potassium (K)	5.63	0.41	4.80	0.55	5.91	0.51	5.83	0.53	**0.009**	0.039	**0.0001**	**0.021**
Sodium (Na)	143.8	2.3	142.4	4.2	147.0	1.0	148.8	2.2	0.326	**0.049**	**0.031**	0.116
Iron (Fe), μmol L^−1^	19.6	8.1	32.7	7.8	26.9	4.7	38.4	12.5	**0.0001**	0.066	0.165	0.124
Calcium (Ca), nmol L^−1^	2.69	0.11	2.67	0.11	2.74	0.14	2.79	0.19	**0.008**	0.087	0.116	0.287
Vitamin B12, pmol L^−1^	363.7	106.4	225.0	147.5	179.6	57.0	206.2	82.9	**0.028**	**0.023**	0.664	0.217
Creatinine (CREA), μmol L^−1^	76.6	14.7	96.7	57.5	69.0	9.38	60.0	3.9	0.502	0.133	0.357	0.103
Alkaline phosphatase (AP), U L^−1^	262.3	83.9	309.3	42.3	259.4	40.6	235.6	31.4	0.190	0.918	**0.007**	**0.047**
Urea, mmol L^−1^	2.44	0.95	2.32	0.92	3.3	0.6	2.44	0.44	**0.032**	0.058	0.713	**0.009**

AV, average; SD, standard deviation; C, control group, fed with the basal diet; T, treated group, fed with the fermented feed; 25 d, 25-day-old piglets; 61 d, 61-day-old piglets.

Data are expressed as the mean ± standard deviation (n = 10).

*Data were statistically compared with the paired t-test and column statistics; p ≤ 0.05 was considered significant (bold font)*.

#### Piglet Growth Performance

**Figure 6:**
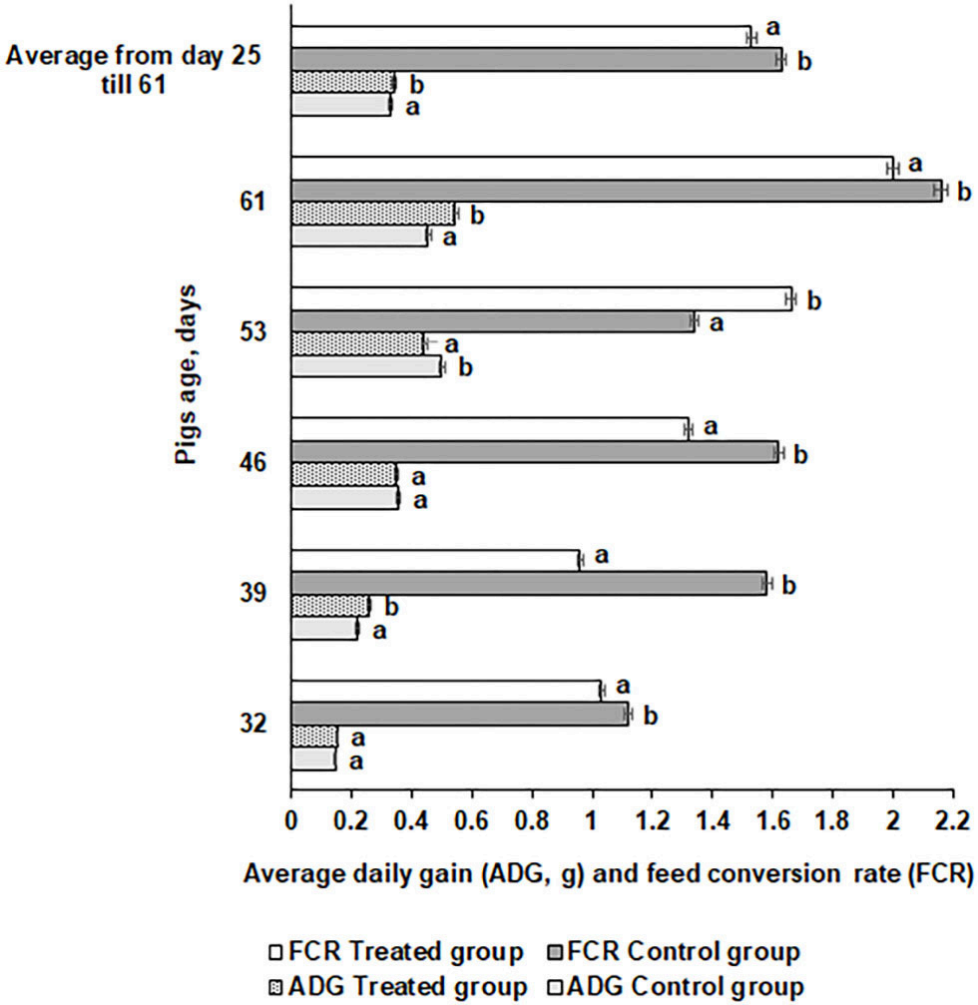
Average daily gain (ADG) and feed conversion rate (FCR) for the pigs. Data are expressed as the mean ± standard deviation (*n* = 10). Data were statistically compared with a paired *t*-test and column statistics. Means followed by different letters are significantly different (*p* ≤ 0.05).

Average daily gain (ADG) and FE for the piglets are shown in Figure 6. The ADG was significantly higher at day 61 in the treated compared with the control piglets (0.546 vs. 0.455 kg, respectively). However, there were distinct tendencies during the different time points. At day 46, there was no difference in ADG between the groups, while at day 53, the control group ADG was higher than the treated group. In most of the cases, FE was higher in the control group piglets (except at day 53) compared with the treated group (1.63 and 1.53 kg, respectively). In modern swine production, fermented feed has been included to reduce the use of antibiotic growth promoters (63) and decrease feed price by using food processing by-products (64). During the fermentation process, most of the antinutritional factors are degraded, macronutrients are converted to lower-molecular-weight and more digestible compounds, and probiotics and their desirable metabolites occur in fermentable substrate (47, 65). The use of feed with a high content of viable desirable microorganisms increases the bioavailability of feed and improves pigs' digestibility and overall gastrointestinal functions (44), reduces the risk of diarrhea (66), and benefits pigs' health and growth performance (67). The use of fermented feed can reduce the feed cost in animal production (68). Supplementation with fermented feed increases organic acids and short-chain fatty acid concentrations in the hindgut (49) and improves intestinal functions, all of which further improve performance (69). Finally, feed fermentation with selected starters increases the nutritional quality and utilization of feed and provides health-related microorganisms that exert growth-promoting effects in the animals.

#### *In vivo* Mycotoxins Bioconversion

The mycotoxin concentration (μg kg^−1^) in feed (basal and fermented) and piglets' fecal samples (control and treated groups on days 25 and 61) is shown in Table 4. AME was found in day 61 control feces and in fermented feed samples. However, AME was not found in feces from treated pigs. *Alternaria* fungi produce many secondary metabolites (more than 70); however, the most known are AOH, AME, tenuazonic acid (TeA), and TNX (70). While AME is mutagenic *in vitro*, there is limited evidence for AME carcinogenicity (71). AME has strong antifungal activity (72, 73) and exhibits genotoxic potential (74). Notably, AME is readily hydroxylated by pig hepatic microsomes (74). While AME is genotoxic at high micromolar concentrations *in vitro* (75), the cumulative concentration at the highest applied extract dose was calculated to be 54 nM, ~1,000-fold lower than what would be needed to impair DNA integrity (76).

**Table 4:** Mycotoxin concentrations (μg/kg) in feed and fecal samples at days 25 and 61.

**Samples**	**Mycotoxin concentration**, **μg/kg**					
	**AME**	**ALT**	**FB1**	**ROQ-C**	**TNX**	**15-AcDON**
**Control group feed and piglets' fecal samples**						
Basal feed	–	–	–	–	25.4 ± 2.5	–
C25d	–	–	–	–	–	–
C61d	8.8 ± 1.2	19.2 ± 1.8	–	–	–	–
**Treated group feed and piglets' fecal samples**						
Treated feed	17.06 ± 1.1	10.1 ± 0.9	–	16.4 ± 1.2	109.7 ± 4.6	66.7 ± 3.9
T25d	–	–	58.1 ± 2.5	–	–	–
T61d	–	–	34.9 ± 1.9	–	22.4 ± 3.1	–

AME, alternariol monomethyl ether; ALT, altenuene; FB1, fumonisin B1; ROQ-C, roquefortine-C; TNX, tentoxin; 15-AcDON, 15-acetyldeoxynivalenol.

C, control group, fed with the basal diet; T, treated group, fed with the fermented feed; 25 d, 25-day-old piglets; 61 d–61-day-old piglets.

*Data for the feed samples are expressed as the mean ± standard deviation (n = 3). Data for the fecal samples are expressed as the mean ± standard deviation (n = 10)*.

Another secondary metabolite, ALT, showed a similar tendency to AME in the tested samples. Specifically, ALT was found in day 61 control group piglets' feces and in fermented feed samples. Alternaria mycotoxins contaminate cereal and may impact animal health, but data on its mammalian metabolism are scarce (77). The Alternaria mycotoxins often contaminate feed, a phenomenon that leads to a challenge for risk assessment. Some Alternaria mycotoxins possess estrogenic properties, which, together with other compounds such as ALT, iso-altenuene (iso-ALT), or altenuisol (ATL), can form the dibenzo-α-pyrone group of Alternaria toxins. Synergistic and cumulative effects might increase the toxicological effect of separate compounds. Thus, it is unclear which mechanism of action exerts a significant impact for adverse outcomes. This effect particularly applies to the estrogenic activity of dibenzo-α-pyrones, as growth-stimulating effects triggered by endocrine disruption are obviously only of potential relevance in sub-cytotoxic and sub-genotoxic concentrations. Finally, numerous *Alternaria* produce large quantities and varieties of toxins. Given this diverse mixture, it might lead to an overlay of distinct bioactivities depending on the qualitative and quantitative compositions of the exposure. The naturally occurring composition of *Alternaria* toxins might contain contrary effects and possess weak estrogenic activity (e.g., AOH, AME, and their respective metabolites) or anti-estrogenic activity (76).

FB1 is the most abundant and documented fumonisin toxin; it is produced by more than 30 species (78). FB1 is nephrotoxic and hepatotoxic (79, 80) and exhibits deleterious effects on animal health (81). Other clinical diseases induced by FB1 are leukoencephalomalacia, pulmonary edema, cardiac dysfunction (82), carcinogenesis (83), neural tube defects (84), and disruption of the intestines and immune system (85). Contamination levels of FB1 in feed are strictly regulated (86, 87). However, the mechanisms associated with FB1 toxicity remain unclear (81). FB1 reduces the concentration of ceramide and sphingomyelin and increases the levels of Sa and sphingolipid terminal products. For this reason, the Sa/So ratio is called a biomarker of FB1 exposure in animals (88, 89). There is a correlation between sphingolipids and the changes of other lipids (sterols and fatty acids). In the adipose tissue of rats, Cers4 is a potent target of endogenous lipid metabolism modulators (90), insulin, and/or changes in phospholipid transfer protein activity (91). Moreover, FA elongase 1 activity, which is included in both saturated and monounsaturated FA synthesis, is regulated by sphingolipid metabolism products (92). Sphingomyelins are also included in the post-translational processes of master regulators of FA and cholesterol metabolism (93). Kinome and transcriptome profiles of piglets exposed to FB1 showed that most of the effects of the mycotoxin are mediated by the influence on ceramide concentration (94). This mechanism of action induces the reduction of integrin-mediated cell-matrix adhesion, an inflammatory response, and alters the expression of genes included in cholesterol and FA homeostasis (95).

ROQ-C is a typical mycotoxin for Northern and Western European countries. It is frequently found in grass silages (96). A concentration of 25,000 μg kg^−1^ has no toxicological effect on sheep (97). However, there is a relationship between dairy cow diseases (paralysis, ketosis, and inappetence) and ROQ-C levels in feed at 25,000 μg kg^−1^ (98). Pigs are very sensitive to mycotoxins. Due to their high consumption of cereals, pigs are exposed to these toxins, as well as chronic contamination. Mycotoxins modulate the immune response of pigs, an action that leads to non-resistance to infectious diseases and lower vaccine efficacy. Furthermore, mycotoxins indirectly affect animal productivity (99). In EU, six mycotoxins, as feed contaminants, are reglemented: aflatoxins (AF), OTA, fumonisins (FB), ZEN, and trichothecenes (principally DON, T-2 and HT-2 toxins) (100). Notably, ROQ-C is not on the regulated mycotoxins list.

TNX was found in both basal and fermented feed (25.4 and 109.7 μg kg^−1^, respectively), as well as in 61-day-old treated piglet feces (22.4 μg kg^−1^). Till now, there are no regulations on *Alternaria* toxins in feed. AOH, AME, TeA, iso-TaA, ATXs, tentoxin (TEN), and ALT have been identified and chemically characterized. By increasing the sensitivity of the analytic techniques, several other *Alternaria* toxins have been identified. Information about *Alternaria* toxins in feed, their changes during the technological processes, and other factors is scarce. There is no information published about absorption, distribution, and excretion of *Alternaria* mycotoxins in animals. It was published that TEN is not mutagenic in bacteria. However, no data are available for *Alternaria* toxins, including *in vivo* genotoxicity or carcinogenicity. There is not enough knowledge about the possible effects of *Alternaria* toxins on farm, as well as about the occurrence of these mycotoxins in feed and, for this reason, to assess the risk regarding *Alternaria* toxins for animal health is not possible (101). However, the presence of emerging, masked, modified, etc. mycotoxins revealed by new analytical methods can also increase the health risk for pigs. Currently, very few studies document the occurrence and toxicity of these toxins. Finally, there is a need to determine the risk they represent in pig production (102).

Another compound, 15-AcDON, was found only in fermented feed samples (66.7 μg kg^−1^). Various factors can alter a mycotoxin's chemical structure. One of the factors is their hydrolysis to free DON or deepoxy-deoxynivalenol (DOM-1) by the intestinal microflora (103). In animals, mycotoxin metabolic detoxification has been described as the deep oxidation of DON, with the participation of intestinal microflora (102, 104) to DOM-1. This mechanism occurs in pigs and other animals. Furthermore, during DON biotransformation, it is conjugated with glucuronides, sulfonates, or glutathione (105). Glucuronidation involves UDP-glucuronosyltransferase activity and can occur in intestinal microsomes (104). Epoxidation is not very significant in pigs; however, glucuronidation is a very important factor of DON metabolism (106, 107). Conjugation of mycotoxins in animals contribute to the formation of DON-3,8,15-glucuronides (107). Enzyme-catalyzed glucuronidation is a slow process that is strongly influenced on the animal species (108). Modified DON forms are scarcely reported. European regulations limit the maximum permissible levels of major mycotoxins in feed also on animal age (for example, 900–12,000 μg kg^−1^ for DON) (109, 110). Finally, the risk for animals to be influenced by modified mycotoxins may be very high, and it is very important to start DON analog regulation in feed. *Fusarium* fungi that produce DON are separated into two sub-groups, according to chemotype: 3Ac-DON and 15-AcDON, these chemotypes may generate acetylated derivatives (111). In 2010, the Expert Committee of FAO/WHO for food additives published that acetyl derivatives of DON, also, should be controlled. In 2017, the European Food Safety Authority published report on hazards for animals by DON and its acetylated/modified derivatives in feed, where cereals are mentioned as the main risk source. Recent studies published that safety/toxicity of masked mycotoxins highly depends on the toxin type and the exposure (112). It was published that toxicity of the DON and its acetylated derivatives (3Ac-DON and 15Ac-DON) are potentially different. The first barrier for contaminants is an intestinal epithelium, which is highly sensitive to mycotoxins, particularly DON. It was reported that 3Ac-DON is less toxic than DON and that DON is less toxic than 15Ac-DON. The latter compound lowered the protective functions of the intestinal epithelium; however, such an influence of 3Ac-DON and DON on epithelium was not established. These findings were confirmed in *ex vivo* and *in vivo* studies (113).

Finally, mycotoxin biotransformation mechanism can be influenced by many factors (absolute concentration of mycotoxin, mycotoxin profile in intestine, dietary composition, and conditions, etc.), of which the microorganisms profile of the digestive tract is very important (103). Regulation applies only to the parent compounds and, unfortunately, does not include modified forms that are commonly present in feed. Abovementioned forms are a big challenge for the scientific community, namely, because no data are currently available on the toxicity and relations with other mycotoxins *in vivo*.

## Conclusions

Compared to a commercially available LAB composition, the novel LAB composition effectively reduced feed pH, produced a 2-fold higher L(+) lactic acid content, increased the viable LAB count (on average 8.8 log_10_ CFU g^−1^), and led to stable feed fermentation during the 36-day experimental period. Fecal microbiota analysis showed an increased number of probiotic bacteria, particularly *Lactobacillus*, in the treated compared with the control group at the end of experiment. These data indicate that fermented feed can modify microbial profile change in the gut of pigs. Furthermore, fermented feed improved the hematological profile of the treated piglets. Mycotoxin analysis revealed that AME and ALT were found in 61-day-old control group piglets' feces and in fermented feed samples. However, AME was absent from treated piglet feces. Finally, feed fermentation with new LAB strain combination is very promising as a piglet microbiota modulation factor to improve nutrient absorption, growth performance, and health parameters. We also described a promising technology to increase local feed stock uses and make the process more economically feasible.

## Data Availability Statement

All datasets generated for this study are included in the article/Supplementary Material.

## Ethics Statement

All animal procedures were conducted according to the EU Directive (12) of the European Parliament and of Council from 22 September 2010 on the protection of animals used for scientific purposes and Requirements for the Keeping, Maintenance and Use of Animals Intended for Science and Education Purposes, approved by the order of the Lithuanian Director of the State Food and Veterinary Service (31/10/2012, No. B1-866) (13). This study was conducted at a pig farm in the Klaipeda district (Kontvainiai, Lithuania) and at the Institute of Animal Rearing Technologies, Lithuanian University of Health Sciences (Kaunas, Lithuania).

## Author Contributions

The LUHS group conceived the study, participated in its design and coordination, participated in interpretation of the findings, and drafted the manuscript. The KTU group participated in the animal studies, statistical analysis, and interpretation of the findings in the manuscript. The BIOR group developed the method for mycotoxin (including masking mycotoxins) determination in feed and fecal samples and performed the analysis. All authors (LV, MR, VL, VS, PZ, EZ, VB, IP, IR, SB, DK, EM, AD, RG, and EB) read and approved the final manuscript.

## Conflict of Interest

The authors declare that the research was conducted in the absence of any commercial or financial relationships that could be construed as a potential conflict of interest.

## Supplementary Material

Supplementary File 1 Control group before experiment genera.

Supplementary File 2 Experimental group before experiment genera.

Supplementary File 3 Control group after experiment genera.

Supplementary File 4 Experimental group after experiment genera.

Supplementary File 5 Control group after experiment species.

Supplementary File 6 Experimental group after experiment species.
