# Supplementary material for: Combination of Antimicrobial Starters for Feed Fermentation: Influence on Piglet Feces Microbiota and Health and Growth Performance, Including Mycotoxin Biotransformation in vivo
Source: Front Vet Sci. 2020 Oct 16;7:528990. doi: 10.3389/fvets.2020.528990 (PMC7596189; doi:10.3389/fvets.2020.528990)
Supplement: Supplementary File 1 — Control group before experiment genera. [file Data_Sheet_1.PDF]

BaseClear Genome Explorer

Genus	Number of reads	Relative abundance
Prevotella	13372	32.52%
Lactobacillus	3776	9.18%
Clostridium	2759	6.71%
Escherichia	1381	3.35%
Unclassified	1269	3.08%
Alloprevotella	1014	2.46%
Faecalibacterium	991	2.41%
Blautia	831	2.02%
Terrisporobacter	765	1.86%
Eubacterium	729	1.77%
Roseburia	705	1.71%
unclassified Bacteroidales	635	1.54%
Ruminococcus	610	1.48%
Parabacteroides	567	1.37%
Barnesiella	550	1.33%
unclassified Lachnospiraceae	460	1.11%
Lachnoclostridium	433	1.05%
Ruminiclostridium	426	1.03%
Flintibacter	426	1.03%
Gemmiger	392	0.95%
Paraprevotella	374	0.9%
Intestinimonas	366	0.89%
Anaerovibrio	343	0.83%
Bacteroides	339	0.82%
Oscillibacter	328	0.79%
Turicibacter	308	0.74%
Phascolarctobacterium	282	0.68%
Campylobacter	277	0.67%
Romboutsia	275	0.66%
Butyricicoccus	270	0.65%
Oscillospira	227	0.55%
Coprococcus	221	0.53%
unclassified Eubacteriaceae	195	0.47%
Fusicatenibacter	191	0.46%
Desulfovibrio	188	0.45%
Intestinibacter	181	0.44%
Dorea	149	0.36%
Catenibacterium	146	0.35%
Sporobacter	139	0.33%
Collinsella	135	0.32%
Murimonas	112	0.27%
Candidatus Soleaferrea	112	0.27%
Treponema	110	0.26%
Butyrivibrio	100	0.24%

Genus	Number of reads	Relative abundance
Holdemanella	98	0.23%
unclassified Deltaproteobacteria	90	0.21%
Acetivibrio	88	0.21%
Enorma	82	0.19%
Bifidobacterium	82	0.19%
Paludibacter	80	0.19%
Olsenella	78	0.18%
Fournierella	76	0.18%
Kluyvera	73	0.17%
Tannerella	72	0.17%
Erysipelothrix	72	0.17%
Saccharofermentans	66	0.16%
unclassified Prevotellaceae	63	0.15%
Anaerotaenia	63	0.15%
unclassified Clostridiales	63	0.15%
Porphyromonas	61	0.14%
Hungatella	55	0.13%
unclassified Clostridiales Family XIII. Incertae Sedis	55	0.13%
unclassified Erysipelotrichaceae	54	0.13%
Acetanaerobacterium	52	0.12%
Anaerostipes	48	0.11%
Olivibacter	46	0.11%
Falcatimonas	46	0.11%
Tyzzerella	46	0.11%
Solobacterium	45	0.1%
Sutterella	42	0.1%
Anaerobacterium	41	0.09%
Ruthenibacterium	40	0.09%
Holdemania	40	0.09%
Agathobacter	38	0.09%
Vallitalea	38	0.09%
Gracilibacter	37	0.09%
Denitrobacterium	36	0.08%
Mucispirillum	36	0.08%
Lachnospira	36	0.08%
unclassified Mollicutes	34	0.08%
Herbinix	34	0.08%
Shigella	32	0.07%
Enterorhabdus	32	0.07%
Asaccharospora	32	0.07%
unclassified Ruminococcaceae	31	0.07%
Candidatus Glomeribacter	31	0.07%
Anaerovorax	30	0.07%
Paeniclostridium	29	0.07%
Anaeromassilibacillus	29	0.07%
Christensenella	29	0.07%
Anaerobium	29	0.07%

Genus	Number of reads	Relative abundance
Mogibacterium	28	0.06%
Oribacterium	27	0.06%
Anaerocolumna	24	0.05%
Pseudoflavonifractor	24	0.05%
Eisenbergiella	24	0.05%
Peptococcus	23	0.05%
unclassified Clostridia	21	0.05%
Natranaerovirga	21	0.05%
Selenomonas	21	0.05%
Papillibacter	20	0.04%
Parasutterella	20	0.04%
Salmonella	19	0.04%
Hespellia	19	0.04%
Succinivibrio	19	0.04%
unclassified Veillonellaceae	18	0.04%
Brassicibacter	18	0.04%
Acidaminobacter	18	0.04%
Rikenella	17	0.04%
Staphylococcus	17	0.04%
Breznakia	17	0.04%
Desulfotomaculum	17	0.04%
Gorbachella	17	0.04%
unclassified Planctomycetales	16	0.03%
Methanobrevibacter	16	0.03%
Caloramator	16	0.03%
Anaeroplasma	16	0.03%
Sphaerochaeta	15	0.03%
Bulleidia	15	0.03%
Geosporobacter	15	0.03%
Catabacter	14	0.03%
unclassified Erysipelotrichia	14	0.03%
Mobilitalea	14	0.03%
Anaerosporobacter	14	0.03%
Oligosphaera	13	0.03%
unclassified Cyanobacteria	13	0.03%
Erysipelatoclostridium	13	0.03%
unclassified Thermoplasmata	13	0.03%
Kosakonia	12	0.02%
Actinobacillus	12	0.02%
Butyricimonas	12	0.02%
Robinsoniella	12	0.02%
unclassified Peptostreptococcaceae	12	0.02%
Slackia	12	0.02%
Flavonifractor	11	0.02%
Acholeplasma	11	0.02%
Bacillus	11	0.02%
Faecalicoccus	11	0.02%

Genus	Number of reads	Relative abundance
unclassified Porphyromonadaceae	10	0.02%
Macellibacteroides	10	0.02%
Lutispora	10	0.02%
Cryptanaerobacter	9	0.02%
Alistipes	9	0.02%
Abyssivirga	9	0.02%
Parvibacter	8	0.01%
Lachnoanaerobaculum	8	0.01%
Coprobacillus	8	0.01%
Candidatus Stoquefichus	8	0.01%
Lactonifactor	7	0.01%
Wautersiella	7	0.01%
Caminicella	7	0.01%
Enterococcus	7	0.01%
Subdoligranulum	7	0.01%
Defluviitalea	7	0.01%
Ethanoligenens	7	0.01%
unclassified Clostridiaceae	7	0.01%
Marvinbryantia	7	0.01%
Thermotalea	6	0.01%
Corynebacterium	6	0.01%
Ruminobacter	6	0.01%
unclassified Betaproteobacteria	6	0.01%
Petrimonas	6	0.01%
Pseudobutyrivibrio	6	0.01%
Pleomorphochaeta	6	0.01%
Anaerotruncus	6	0.01%
Fibrobacter	6	0.01%
Faecalitalea	6	0.01%
Carboxylicivirga	6	0.01%
unclassified Gammaproteobacteria	6	0.01%
Parasporobacterium	6	0.01%
unclassified Bacteroidaceae	6	0.01%
Casaltella	6	0.01%
Proteiniborus	5	0.01%
Photorhabdus	5	0.01%
unclassified Alphaproteobacteria	5	0.01%
Mageeibacillus	5	0.01%
Senegalimassilia	5	0.01%
Megasphaera	5	0.01%
Dysgonomonas	5	0.01%
Helicobacter	4	0%
Caloranaerobacter	4	0%
Asteroleplasma	4	0%
Paraclostridium	4	0%
Alkalibacter	4	0%
Cutibacterium	4	0%

Genus	Number of reads	Relative abundance
unclassified Lactobacillaceae	4	0%
Natronincola	4	0%
unclassified Bacteroidia	3	0%
Mitsuokella	3	0%
Dehalobacterium	3	0%
Serratia	3	0%
Cellulosilyticum	3	0%
Erwinia	3	0%
Candidatus Heliomonas	3	0%
Coprobacter	3	0%
Alkaliphilus	3	0%
Anaerofilum	3	0%
Anaerofustis	3	0%
Propionispira	3	0%
Mycoplasma	3	0%
Acidaminococcus	3	0%
unclassified Bacillales	2	0%
Acetoanaerobium	2	0%
Victivallis	2	0%
Rhodobacter	2	0%
Candidatus Methanomethylophilus	2	0%
Sporanaerobacter	2	0%
Cytophaga	2	0%
Bariatricus	2	0%
Anaerorhabdus	2	0%
Anaerobiospirillum	2	0%
Peptoclostridium	2	0%
Franconibacter	2	0%
Hathewaya	2	0%
Syntrophococcus	2	0%
unclassified Actinobacteria	2	0%
unclassified Paenibacillaceae	2	0%
Caproiciproducens	2	0%
Sphingobacterium	2	0%
Tepidibacter	2	0%
Howardella	2	0%
Citrobacter	2	0%
Clostridioides	2	0%
Lachnobacterium	2	0%
Oceanirhabdus	2	0%
Oxalobacter	2	0%
Mucilaginibacter	2	0%
Desulfosporosinus	1	0%
unclassified Oscillospiraceae	1	0%
Aeromonas	1	0%
Parapedobacter	1	0%
Ercella	1	0%

Genus	Number of reads	Relative abundance
Acetitomaculum	1	0%
Xenorhabdus	1	0%
Massilia	1	0%
Catonella	1	0%
unclassified Streptococcaceae	1	0%
unclassified Candidatus Saccharibacteria	1	0%
Eggerthella	1	0%
Conexibacter	1	0%
Mesoplasma	1	0%
Proteocatella	1	0%
Paenibacillus	1	0%
unclassified Pseudomonadaceae	1	0%
Natranaerobius	1	0%
Vagococcus	1	0%
Tepidimicrobium	1	0%
Dethiobacter	1	0%
Marinilabilia	1	0%
unclassified Enterobacteriaceae	1	0%
Lactococcus	1	0%
Desulfomonile	1	0%
Acetatifactor	1	0%
Streptomyces	1	0%
Herbaspirillum	1	0%
Oxobacter	1	0%
Modestobacter	1	0%
Tindallia	1	0%
Wolinella	1	0%
Fucophilus	1	0%
Actinocorallia	1	0%
Fastidiosipila	1	0%
Dethiosulfatibacter	1	0%
unclassified Chlamydiia	1	0%
Gordonibacter	1	0%
Methanosphaera	1	0%
Pediococcus	1	0%
Moorella	1	0%
Pyramidobacter	1	0%
Proteinivorax	1	0%
Gottschalkia	1	0%
unclassified Erysipelotrichales	1	0%
Desulfonatronum	1	0%
Streptococcus	1	0%
Pseudocitrobacter	1	0%
Candidatus Izimaplasma	1	0%
Acidobacterium	1	0%
Sporocytophaga	1	0%
Odoribacter	1	0%

Genus	Number of reads	Relative abundance
Propionibacterium	1	0%
Atopobium	1	0%
Lysinibacillus	1	0%
Pantoea	1	0%
