# Supplementary material for: Combination of Antimicrobial Starters for Feed Fermentation: Influence on Piglet Feces Microbiota and Health and Growth Performance, Including Mycotoxin Biotransformation in vivo
Source: Front Vet Sci. 2020 Oct 16;7:528990. doi: 10.3389/fvets.2020.528990 (PMC7596189; doi:10.3389/fvets.2020.528990)
Supplement: Supplementary File 2 — Experimental group before experiment genera. [file Data_Sheet_2.PDF]

BaseClear Genome Explorer

Genus	Number of reads	Relative abundance
Prevotella	14919	25.21%
Barnesiella	7191	12.15%
Alloprevotella	5529	9.34%
Bacteroides	3967	6.7%
Unclassified	2223	3.75%
unclassified Bacteroidales	2186	3.69%
Faecalibacterium	1705	2.88%
Parabacteroides	1466	2.47%
Collinsella	1252	2.11%
Oscillospira	1041	1.75%
Flintibacter	1039	1.75%
Tannerella	980	1.65%
Desulfovibrio	932	1.57%
Escherichia	742	1.25%
Paraprevotella	702	1.18%
Intestinimonas	680	1.14%
Gemmiger	647	1.09%
Lactobacillus	571	0.96%
Clostridium	551	0.93%
Catenibacterium	518	0.87%
Denitrobacterium	513	0.86%
Ruminiclostridium	497	0.84%
Sphaerochaeta	485	0.81%
Oscillibacter	473	0.79%
Porphyromonas	384	0.64%
Sporobacter	364	0.61%
Akkermansia	355	0.6%
Megasphaera	344	0.58%
Butyricimonas	333	0.56%
Caloramator	310	0.52%
Fournierella	284	0.48%
Eubacterium	254	0.42%
Gracilibacter	251	0.42%
Treponema	223	0.37%
Sharpea	222	0.37%
Anaerovibrio	206	0.34%
Olivibacter	204	0.34%
Mitsuokella	170	0.28%
Sutterella	161	0.27%
Lachnoclostridium	156	0.26%
Butyricicoccus	149	0.25%
Mucispirillum	137	0.23%
Olsenella	132	0.22%
Blautia	124	0.2%

Genus	Number of reads	Relative abundance
unclassified Planctomycetales	118	0.19%
Oligosphaera	116	0.19%
Candidatus Soleaferrea	101	0.17%
unclassified Prevotellaceae	98	0.16%
Ruminococcus	97	0.16%
unclassified Lachnospiraceae	97	0.16%
unclassified Deltaproteobacteria	92	0.15%
Roseburia	91	0.15%
Acetanaerobacterium	85	0.14%
unclassified Ruminococcaceae	79	0.13%
Christensenella	78	0.13%
Acidaminobacter	73	0.12%
Desulfotomaculum	71	0.12%
unclassified Porphyromonadaceae	71	0.12%
Fusicatenibacter	69	0.11%
Papillibacter	68	0.11%
Enorma	68	0.11%
Rikenella	65	0.1%
Pseudoflavonifractor	61	0.1%
Fibrobacter	59	0.09%
Selenomonas	57	0.09%
unclassified Clostridiales	57	0.09%
unclassified Erysipelotrichaceae	57	0.09%
Geosporobacter	50	0.08%
Cloacibacillus	48	0.08%
Paludibacter	43	0.07%
Slackia	41	0.06%
Coprococcus	41	0.06%
Saccharofermentans	40	0.06%
Ruthenibacterium	38	0.06%
Dorea	38	0.06%
Phascolarctobacterium	36	0.06%
Lutispora	34	0.05%
Candidatus Heliomonas	33	0.05%
Pyramidobacter	33	0.05%
Eggerthella	33	0.05%
Acetivibrio	30	0.05%
Asteroleplasma	30	0.05%
Enterorhabdus	30	0.05%
Parvibacter	29	0.04%
Flavonifractor	28	0.04%
Campylobacter	28	0.04%
Gordonibacter	26	0.04%
Murimonas	26	0.04%
Anaeromassilibacillus	26	0.04%
Intestinibacter	25	0.04%
Kluyvera	24	0.04%

Genus	Number of reads	Relative abundance
Staphylococcus	22	0.03%
unclassified Clostridia	22	0.03%
Succinivibrio	22	0.03%
Alistipes	21	0.03%
Tyzzerella	20	0.03%
unclassified Eubacteriaceae	20	0.03%
Pleomorphochaeta	19	0.03%
Salmonella	19	0.03%
Rarimicrobium	18	0.03%
Butyrivibrio	16	0.02%
Holdemania	16	0.02%
Thermotalea	15	0.02%
Hungatella	15	0.02%
unclassified Clostridiales Family XIII. Incertae Sedis	15	0.02%
Natranaerovirga	15	0.02%
Parasutterella	15	0.02%
unclassified Bacteroidia	14	0.02%
unclassified Verrucomicrobiaceae	14	0.02%
unclassified Spirochaetia	14	0.02%
unclassified Bacteroidaceae	13	0.02%
Paraeggerthella	13	0.02%
Spirochaeta	13	0.02%
Anaerovorax	13	0.02%
Mogibacterium	12	0.02%
Falcatimonas	12	0.02%
Terrisporobacter	12	0.02%
Caloranaerobacter	12	0.02%
unclassified Actinobacteria	12	0.02%
Anaerobium	11	0.01%
Subdoligranulum	11	0.01%
Shigella	11	0.01%
Catabacter	10	0.01%
unclassified Betaproteobacteria	10	0.01%
Bifidobacterium	10	0.01%
Fusobacterium	10	0.01%
Erysipelothrix	9	0.01%
Holdemanella	9	0.01%
Agathobacter	9	0.01%
Cutibacterium	9	0.01%
Dysgonomonas	9	0.01%
Coprobacter	9	0.01%
Macellibacteroides	9	0.01%
Caminicella	9	0.01%
unclassified Clostridiaceae	9	0.01%
Turicibacter	9	0.01%
Caproiciproducens	9	0.01%
Acetatifactor	9	0.01%

Genus	Number of reads	Relative abundance
Oribacterium	8	0.01%
unclassified Chlamydiia	8	0.01%
Thermanaerovibrio	8	0.01%
Anaerotaenia	7	0.01%
Corynebacterium	7	0.01%
Herbaspirillum	7	0.01%
Peptococcus	6	0.01%
Alkalibacter	6	0.01%
Adlercreutzia	6	0.01%
Candidatus Glomeribacter	6	0.01%
Gorbachella	6	0.01%
Cytophaga	6	0.01%
Bittarella	5	0%
Kosakonia	5	0%
Acidaminococcus	5	0%
unclassified Victivallaceae	5	0%
unclassified Gammaproteobacteria	5	0%
Anaerostipes	5	0%
Synergistes	5	0%
Mobilitalea	5	0%
Anaerocolumna	5	0%
unclassified Mollicutes	5	0%
unclassified Veillonellaceae	4	0%
Anaeroplasma	4	0%
Lachnospira	4	0%
unclassified Cyanobacteria	4	0%
Defluviitalea	4	0%
Caldicoprobacter	4	0%
Lachnoanaerobaculum	4	0%
Romboutsia	4	0%
Anaerobacterium	3	0%
Asaccharospora	3	0%
Victivallis	3	0%
Anaerotruncus	3	0%
Anaerofilum	3	0%
Vallitalea	3	0%
Coprobacillus	3	0%
Propionibacterium	3	0%
Garciella	3	0%
Sanguibacteroides	3	0%
Abyssivirga	3	0%
Senegalimassilia	3	0%
Candidatus Vestibaculum	3	0%
Eisenbergiella	3	0%
Wautersiella	2	0%
Cronobacter	2	0%
Methanobrevibacter	2	0%

Genus	Number of reads	Relative abundance
Listeria	2	0%
Schwartzia	2	0%
Pantoea	2	0%
Ethanoligenens	2	0%
Snodgrassella	2	0%
Hespellia	2	0%
Anaerosporobacter	2	0%
Helicobacter	2	0%
Tepidimicrobium	2	0%
unclassified Coriobacteriaceae	2	0%
Falsiporphyromonas	2	0%
Tindallia	2	0%
unclassified Thermoplasmata	2	0%
Moorella	2	0%
Magnetococcus	2	0%
Alkaliflexus	2	0%
Gabonibacter	2	0%
unclassified Bacillales	1	0%
Acetobacteroides	1	0%
Leuconostoc	1	0%
Proteocatella	1	0%
Herbinix	1	0%
Petrimonas	1	0%
Leadbetterella	1	0%
Rothia	1	0%
Sporanaerobacter	1	0%
Geoalkalibacter	1	0%
Granulicatella	1	0%
Streptomyces	1	0%
Odoribacter	1	0%
Paeniclostridium	1	0%
Catenisphaera	1	0%
Pelomonas	1	0%
Anaerobiospirillum	1	0%
Hallella	1	0%
Agrobacterium	1	0%
Parasporobacterium	1	0%
Ruminobacter	1	0%
Heliobacillus	1	0%
Neisseria	1	0%
Nubsella	1	0%
Aminobacterium	1	0%
Erwinia	1	0%
Fusibacter	1	0%
Parapedobacter	1	0%
Caldibacillus	1	0%
unclassified Firmicutes	1	0%

Genus	Number of reads	Relative abundance
Bulleidia	1	0%
Aureivirga	1	0%
Robinsoniella	1	0%
Flavobacterium	1	0%
Actinocorallia	1	0%
Dethiosulfatibacter	1	0%
Stomatobaculum	1	0%
Candidatus Stoquefichus	1	0%
Dielma	1	0%
unclassified Enterobacteriaceae	1	0%
Ureibacillus	1	0%
Edaphobacillus	1	0%
Zoogloea	1	0%
Pseudomonas	1	0%
Neochlamydia	1	0%
Actinomyces	1	0%
Bacillus	1	0%
Hydrogenoanaerobacterium	1	0%
Bordetella	1	0%
Thermomicrobium	1	0%
Pelobacter	1	0%
Nocardioides	1	0%
Sporotomaculum	1	0%
Sphingobacterium	1	0%
Bhargavaea	1	0%
Prolixibacter	1	0%
Flexibacter	1	0%
Sporocytophaga	1	0%
Cupriavidus	1	0%
unclassified Peptostreptococcaceae	1	0%
Faecalicoccus	1	0%
Klebsiella	1	0%
Alkalibaculum	1	0%
Streptococcus	1	0%
unclassified Bacilli	1	0%
Novosphingobium	1	0%
Thermoflavimicrobium	1	0%
unclassified Actinomycetales	1	0%
Faecalitalea	1	0%
unclassified Alphaproteobacteria	1	0%
Eikenella	1	0%
Desulfurispora	1	0%
Geobacter	1	0%
unclassified Polyangiaceae	1	0%
Serratia	1	0%
Thermovirga	1	0%
Stenotrophomonas	1	0%

Genus	Number of reads	Relative abundance
Persicitalea	1	0%
Marvinbryantia	1	0%
Actinobacillus	1	0%
Lysinibacillus	1	0%
Enterococcus	1	0%
Cellulomonas	1	0%
