# Supplementary material for: Combination of Antimicrobial Starters for Feed Fermentation: Influence on Piglet Feces Microbiota and Health and Growth Performance, Including Mycotoxin Biotransformation in vivo
Source: Front Vet Sci. 2020 Oct 16;7:528990. doi: 10.3389/fvets.2020.528990 (PMC7596189; doi:10.3389/fvets.2020.528990)
Supplement: Supplementary File 3 — Control group after experiment genera. [file Data_Sheet_3.PDF]

# BaseClear Genome Explorer

| Genus | Number of reads | Relative abundance |
|---|---|---|
| Prevotella | 23636 | 38.69% |
| Unclassified | 2458 | 4.02% |
| Faecalibacterium | 2403 | 3.93% |
| Roseburia | 2102 | 3.44% |
| Terrisporobacter | 1943 | 3.18% |
| Blautia | 1862 | 3.04% |
| Clostridium | 1740 | 2.84% |
| Gemmiger | 1584 | 2.59% |
| Eubacterium | 1305 | 2.13% |
| Megasphaera | 1073 | 1.75% |
| Parabacteroides | 996 | 1.63% |
| Barnesiella | 985 | 1.61% |
| Ruminococcus | 888 | 1.45% |
| Lactobacillus | 856 | 1.4% |
| Dialister | 834 | 1.36% |
| Collinsella | 756 | 1.23% |
| Olsenella | 739 | 1.2% |
| Alloprevotella | 736 | 1.2% |
| unclassified Lachnospiraceae | 713 | 1.16% |
| Romboutsia | 628 | 1.02% |
| Lachnoclostridium | 590 | 0.96% |
| Flintibacter | 557 | 0.91% |
| Butyricicoccus | 539 | 0.88% |
| Oscillibacter | 530 | 0.86% |
| Enorma | 471 | 0.77% |
| Intestinimonas | 454 | 0.74% |
| Anaerovibrio | 420 | 0.68% |
| unclassified Bacteroidales | 419 | 0.68% |
| Coprococcus | 417 | 0.68% |
| Oscillospira | 408 | 0.66% |
| Ruminiclostridium | 389 | 0.63% |
| Sporobacter | 386 | 0.63% |
| Fusicatenibacter | 377 | 0.61% |
| Mitsuokella | 335 | 0.54% |
| Enterorhabdus | 328 | 0.53% |
| Intestinibacter | 276 | 0.45% |
| Catenibacterium | 275 | 0.45% |
| Dorea | 260 | 0.42% |
| Murimonas | 256 | 0.41% |
| Paraprevotella | 218 | 0.35% |
| Treponema | 183 | 0.29% |
| Desulfovibrio | 170 | 0.27% |
| Phascolarctobacterium | 169 | 0.27% |
| Bacteroides | 143 | 0.23% |

| Genus | Number of reads | Relative abundance |
|---|---|---|
| Butyrivibrio | 134 | 0.21% |
| Acetivibrio | 134 | 0.21% |
| Denitrobacterium | 132 | 0.21% |
| Fournierella | 130 | 0.21% |
| Anaerotaenia | 125 | 0.2% |
| Holdemanella | 108 | 0.17% |
| Turicibacter | 91 | 0.14% |
| Lachnospira | 87 | 0.14% |
| Tannerella | 85 | 0.13% |
| unclassified Prevotellaceae | 79 | 0.12% |
| unclassified Cyanobacteria | 72 | 0.11% |
| Solobacterium | 71 | 0.11% |
| Oribacterium | 70 | 0.11% |
| Anaerovorax | 68 | 0.11% |
| Mogibacterium | 67 | 0.1% |
| Macellibacteroides | 65 | 0.1% |
| unclassified Clostridiales | 63 | 0.1% |
| Falcatimonas | 62 | 0.1% |
| Methanosphaera | 61 | 0.09% |
| Selenomonas | 60 | 0.09% |
| Anaerostipes | 58 | 0.09% |
| unclassified Erysipelotrichaceae | 58 | 0.09% |
| unclassified Ruminococcaceae | 57 | 0.09% |
| Gracilibacter | 54 | 0.08% |
| unclassified Deltaproteobacteria | 52 | 0.08% |
| Paeniclostridium | 52 | 0.08% |
| Asteroleplasma | 52 | 0.08% |
| Hespellia | 50 | 0.08% |
| Erysipelothrix | 50 | 0.08% |
| Asaccharospora | 49 | 0.08% |
| Anaerobacterium | 49 | 0.08% |
| Hungatella | 46 | 0.07% |
| Lutispora | 45 | 0.07% |
| Slackia | 44 | 0.07% |
| Natranaerovirga | 43 | 0.07% |
| Acetanaerobacterium | 42 | 0.06% |
| Christensenella | 42 | 0.06% |
| Sutterella | 42 | 0.06% |
| Candidatus Soleaferrea | 40 | 0.06% |
| Porphyromonas | 38 | 0.06% |
| Anaerobium | 38 | 0.06% |
| Eisenbergiella | 38 | 0.06% |
| Lachnoanaerobaculum | 37 | 0.06% |
| Staphylococcus | 36 | 0.05% |
| Ruthenibacterium | 36 | 0.05% |
| Vallitalea | 35 | 0.05% |
| Pseudoflavonifractor | 35 | 0.05% |

| Genus | Number of reads | Relative abundance |
|---|---|---|
| Saccharofermentans | 35 | 0.05% |
| Gorbachella | 33 | 0.05% |
| Rikenella | 33 | 0.05% |
| Subdoligranulum | 32 | 0.05% |
| Anaeromassilibacillus | 32 | 0.05% |
| Papillibacter | 31 | 0.05% |
| unclassified Eubacteriaceae | 31 | 0.05% |
| Holdemania | 30 | 0.04% |
| Peptococcus | 29 | 0.04% |
| Parvibacter | 26 | 0.04% |
| Agathobacter | 23 | 0.03% |
| Corynebacterium | 23 | 0.03% |
| Escherichia | 23 | 0.03% |
| Paludibacter | 23 | 0.03% |
| Oligosphaera | 23 | 0.03% |
| unclassified Clostridia | 22 | 0.03% |
| Marvinbryantia | 21 | 0.03% |
| Parasutterella | 20 | 0.03% |
| unclassified Mollicutes | 20 | 0.03% |
| Ethanoligenens | 20 | 0.03% |
| Tyzzerella | 18 | 0.02% |
| Allisonella | 18 | 0.02% |
| Abyssivirga | 18 | 0.02% |
| unclassified Planctomycetales | 17 | 0.02% |
| Robinsoniella | 16 | 0.02% |
| Geosporobacter | 16 | 0.02% |
| Flavonifractor | 16 | 0.02% |
| Paraeggerthella | 16 | 0.02% |
| unclassified Clostridiales Family XIII. Incertae Sedis | 16 | 0.02% |
| Herbinix | 15 | 0.02% |
| Coprobacillus | 15 | 0.02% |
| unclassified Veillonellaceae | 15 | 0.02% |
| unclassified Peptostreptococcaceae | 15 | 0.02% |
| Anaerocolumna | 14 | 0.02% |
| Clostridioides | 13 | 0.02% |
| Fibrobacter | 13 | 0.02% |
| Caminicella | 13 | 0.02% |
| Natronincola | 13 | 0.02% |
| Sphaerochaeta | 13 | 0.02% |
| Mobilitalea | 12 | 0.01% |
| Paraclostridium | 12 | 0.01% |
| unclassified Porphyromonadaceae | 12 | 0.01% |
| Acidaminobacter | 11 | 0.01% |
| Adlercreutzia | 11 | 0.01% |
| Bacillus | 11 | 0.01% |
| Bifidobacterium | 11 | 0.01% |
| Campylobacter | 10 | 0.01% |

| Genus | Number of reads | Relative abundance |
| --- | --- | --- |
| Cutibacterium | 10 | 0.01% |
| Pseudobutyrivibrio | 9 | 0.01% |
| Wautersiella | 9 | 0.01% |
| Desulfotomaculum | 8 | 0.01% |
| Atopobium | 8 | 0.01% |
| Bulleidia | 8 | 0.01% |
| unclassified Actinobacteria | 8 | 0.01% |
| Brassicibacter | 8 | 0.01% |
| Lactonifactor | 8 | 0.01% |
| Candidatus Heliomonas | 8 | 0.01% |
| Parasporobacterium | 7 | 0.01% |
| Lachnobacterium | 7 | 0.01% |
| Candidatus Stoquefichus | 7 | 0.01% |
| unclassified Thermoplasmata | 7 | 0.01% |
| unclassified Betaproteobacteria | 6 | 0% |
| Cryptanaerobacter | 6 | 0% |
| Caloramator | 6 | 0% |
| Desulfosporosinus | 6 | 0% |
| Gottschalkia | 6 | 0% |
| Bariatricus | 6 | 0% |
| Anaeroplasma | 5 | 0% |
| Caproiciproducens | 5 | 0% |
| Dielma | 5 | 0% |
| Stomatobaculum | 5 | 0% |
| Breznakia | 5 | 0% |
| Acetoanaerobium | 5 | 0% |
| Anaerosporobacter | 5 | 0% |
| Defluviitalea | 5 | 0% |
| Gordonibacter | 4 | 0% |
| Acetatifactor | 4 | 0% |
| Dehalobacterium | 4 | 0% |
| unclassified Bacteroidaceae | 4 | 0% |
| Pleomorphochaeta | 4 | 0% |
| Senegalimassilia | 4 | 0% |
| Anaerofilum | 4 | 0% |
| Helicobacter | 4 | 0% |
| Syntrophococcus | 4 | 0% |
| Erysipelatoclostridium | 4 | 0% |
| Drancourtella | 4 | 0% |
| Proteocatella | 3 | 0% |
| Succinivibrio | 3 | 0% |
| Acidaminococcus | 3 | 0% |
| Catonella | 3 | 0% |
| unclassified Spirochaetia | 3 | 0% |
| Mucispirillum | 3 | 0% |
| Anaerotruncus | 3 | 0% |
| Catabacter | 3 | 0% |

| Genus | Number of reads | Relative abundance |
| --- | --- | --- |
| unclassified Peptococcaceae | 3 | 0% |
| Thermotalea | 3 | 0% |
| Moorella | 3 | 0% |
| unclassified Alphaproteobacteria | 3 | 0% |
| Sphingobium | 2 | 0% |
| Streptococcus | 2 | 0% |
| Oxalobacter | 2 | 0% |
| unclassified Clostridiaceae | 2 | 0% |
| Alistipes | 2 | 0% |
| Dysgonomonas | 2 | 0% |
| Acetitomaculum | 2 | 0% |
| Listeria | 2 | 0% |
| unclassified Erysipelotrichia | 2 | 0% |
| Anaerobiospirillum | 2 | 0% |
| Oceanirhabdus | 2 | 0% |
| Oxobacter | 2 | 0% |
| Anaerococcus | 2 | 0% |
| Peptoclostridium | 2 | 0% |
| Casaltella | 2 | 0% |
| unclassified Victivallaceae | 2 | 0% |
| Haloplasma | 2 | 0% |
| Melghirimyces | 1 | 0% |
| Desulfitobacterium | 1 | 0% |
| Acinetobacter | 1 | 0% |
| Propionibacterium | 1 | 0% |
| unclassified Candidatus Saccharibacteria | 1 | 0% |
| Paraburkholderia | 1 | 0% |
| Brevibacillus | 1 | 0% |
| unclassified Oscillatoriophycideae | 1 | 0% |
| Chelativorans | 1 | 0% |
| Shigella | 1 | 0% |
| Fenollaria | 1 | 0% |
| Anaerorhabdus | 1 | 0% |
| Thermanaerovibrio | 1 | 0% |
| Paenibacillus | 1 | 0% |
| Aeromonas | 1 | 0% |
| Thiobaca | 1 | 0% |
| Alkalibacter | 1 | 0% |
| Pediococcus | 1 | 0% |
| Moraxella | 1 | 0% |
| Propionispira | 1 | 0% |
| Dongia | 1 | 0% |
| Fucophilus | 1 | 0% |
| Snodgrassella | 1 | 0% |
| Actinocorallia | 1 | 0% |
| Candidatus Methanomethylophilus | 1 | 0% |
| Fastidiosipila | 1 | 0% |

| Genus | Number of reads | Relative abundance |
|---|---|---|
| Dethiosulfatibacter | 1 | 0% |
| unclassified Epsilonproteobacteria | 1 | 0% |
| Proteinivorax | 1 | 0% |
| Acholeplasma | 1 | 0% |
| Shuttleworthia | 1 | 0% |
| Cellulosibacter | 1 | 0% |
| Centipeda | 1 | 0% |
| Tepidibacter | 1 | 0% |
| Marinithermofilum | 1 | 0% |
| Candidatus Izimaplasma | 1 | 0% |
| Schlegelella | 1 | 0% |
| Kluyvera | 1 | 0% |
| Sphingoaurantiacus | 1 | 0% |
| unclassified Desulfomicrobiaceae | 1 | 0% |
| Actinomyces | 1 | 0% |
| Pyramidobacter | 1 | 0% |
| Caloranaerobacter | 1 | 0% |
| Magnetococcus | 1 | 0% |
| Bittarella | 1 | 0% |
| unclassified Streptococcaceae | 1 | 0% |
| Schwartzia | 1 | 0% |
| Pelobacter | 1 | 0% |
| unclassified Gammaproteobacteria | 1 | 0% |
| Solitalea | 1 | 0% |
| Petrimonas | 1 | 0% |
| Gardnerella | 1 | 0% |
| Garciella | 1 | 0% |
| Kosakonia | 1 | 0% |
| Lawsonella | 1 | 0% |
| Sellimonas | 1 | 0% |
| Izhakiella | 1 | 0% |
| Faecalicoccus | 1 | 0% |
| Kopriimonas | 1 | 0% |
| Eggerthella | 1 | 0% |
| Catenuloplanes | 1 | 0% |
| Nesterenkonia | 1 | 0% |
| Tepidimicrobium | 1 | 0% |
| Asaccharobacter | 1 | 0% |
| Alkaliphilus | 1 | 0% |
| Kandleria | 1 | 0% |
| Cryptobacterium | 1 | 0% |
| Cellulosilyticum | 1 | 0% |
| Paracoccus | 1 | 0% |
| Cryocola | 1 | 0% |
| unclassified Coriobacteriaceae | 1 | 0% |
| unclassified Lactobacillaceae | 1 | 0% |
| Streptacidiphilus | 1 | 0% |

| Genus | Number of reads | Relative abundance |
|---|---|---|
| Proteus | 1 | 0% |
| Geobacter | 1 | 0% |
| Methyloterrigena | 1 | 0% |
