# Supplementary material for: Combination of Antimicrobial Starters for Feed Fermentation: Influence on Piglet Feces Microbiota and Health and Growth Performance, Including Mycotoxin Biotransformation in vivo
Source: Front Vet Sci. 2020 Oct 16;7:528990. doi: 10.3389/fvets.2020.528990 (PMC7596189; doi:10.3389/fvets.2020.528990)
Supplement: Supplementary File 4 — Experimental group after experiment genera. [file Data_Sheet_4.PDF]

BaseClear Genome Explorer

Genus	Number of reads	Relative abundance
Prevotella	15910	32.86%
Lactobacillus	11460	23.66%
Clostridium	2865	5.91%
unclassified Lachnospiraceae	1416	2.92%
Unclassified	1018	2.1%
Terrisporobacter	931	1.92%
Faecalibacterium	841	1.73%
Streptococcus	816	1.68%
Megasphaera	745	1.53%
Alloprevotella	739	1.52%
Blautia	684	1.41%
Eubacterium	625	1.29%
Sporobacter	588	1.21%
Roseburia	577	1.19%
Gemmiger	569	1.17%
Barnesiella	479	0.98%
Anaerovibrio	479	0.98%
Butyricicoccus	459	0.94%
Ruminococcus	419	0.86%
Parabacteroides	392	0.8%
Ruminiclostridium	354	0.73%
unclassified Bacteroidales	336	0.69%
Lachnoclostridium	318	0.65%
Paraprevotella	252	0.52%
Flintibacter	237	0.48%
Intestinimonas	229	0.47%
Phascolarctobacterium	214	0.44%
Intestinibacter	191	0.39%
Bacteroides	189	0.39%
Butyrivibrio	184	0.38%
Coprococcus	179	0.36%
Fusicatenibacter	145	0.29%
Catenibacterium	136	0.28%
Oscillibacter	133	0.27%
Mitsuokella	125	0.25%
Oscillospira	104	0.21%
Holdemanella	103	0.21%
Romboutsia	102	0.21%
Dorea	99	0.2%
Turicibacter	92	0.19%
Olsenella	80	0.16%
Murimonas	68	0.14%
Collinsella	65	0.13%
Desulfovibrio	61	0.12%

Genus	Number of reads	Relative abundance
Anaerotaenia	57	0.11%
Acetivibrio	54	0.11%
Solobacterium	53	0.1%
Lachnospira	52	0.1%
Candidatus Soleaferrea	51	0.1%
Tannerella	51	0.1%
Anaerostipes	49	0.1%
Vallitalea	48	0.09%
Dialister	47	0.09%
unclassified Cyanobacteria	47	0.09%
Fournierella	46	0.09%
Christensenella	44	0.09%
Agathobacter	44	0.09%
Porphyromonas	43	0.08%
Sutterella	40	0.08%
Brassicibacter	40	0.08%
Treponema	40	0.08%
Paludibacter	39	0.08%
Mogibacterium	38	0.07%
unclassified Erysipelotrichaceae	34	0.07%
unclassified Clostridiales	34	0.07%
Succinivibrio	33	0.06%
Gracilibacter	33	0.06%
Anaerocolumna	32	0.06%
Anaerobacterium	32	0.06%
Hungatella	32	0.06%
Oribacterium	30	0.06%
unclassified Ruminococcaceae	28	0.05%
unclassified Prevotellaceae	27	0.05%
Denitrobacterium	26	0.05%
Staphylococcus	26	0.05%
unclassified Mollicutes	26	0.05%
Acetanaerobacterium	26	0.05%
Erysipelothrix	26	0.05%
Methanosphaera	26	0.05%
Acidaminobacter	25	0.05%
Falcatimonas	25	0.05%
Peptococcus	24	0.04%
Asaccharospora	23	0.04%
Lactococcus	23	0.04%
Selenomonas	22	0.04%
Caloramator	21	0.04%
unclassified Eubacteriaceae	21	0.04%
Papillibacter	21	0.04%
Anaerovorax	21	0.04%
Natranaerovirga	20	0.04%
Saccharofermentans	19	0.03%

Genus	Number of reads	Relative abundance
Parasutterella	19	0.03%
Propionispira	19	0.03%
unclassified Deltaproteobacteria	19	0.03%
Enorma	17	0.03%
unclassified Veillonellaceae	16	0.03%
Holdemania	16	0.03%
Paeniclostridium	16	0.03%
Hespellia	16	0.03%
unclassified Porphyromonadaceae	15	0.03%
Herbinix	15	0.03%
unclassified Streptococcaceae	15	0.03%
Lachnoanaerobaculum	14	0.02%
Desulfotomaculum	14	0.02%
Campylobacter	14	0.02%
Ruthenibacterium	14	0.02%
Anaerobium	13	0.02%
Rikenella	13	0.02%
unclassified Clostridiales Family XIII. Incertae Sedis	12	0.02%
Corynebacterium	12	0.02%
unclassified Peptostreptococcaceae	12	0.02%
Pseudobutyrivibrio	12	0.02%
Bacillus	11	0.02%
Faecalicoccus	11	0.02%
unclassified Clostridia	11	0.02%
Tyzzerella	11	0.02%
Acidaminococcus	10	0.02%
unclassified Thermoplasmata	10	0.02%
Enterococcus	10	0.02%
Gorbachella	10	0.02%
Enterorhabdus	10	0.02%
Ethanoligenens	10	0.02%
Abyssivirga	9	0.01%
Pseudoflavonifractor	9	0.01%
Coprobacillus	9	0.01%
Helicobacter	9	0.01%
Slackia	8	0.01%
Subdoligranulum	8	0.01%
Lutispora	8	0.01%
Anaeromassilibacillus	8	0.01%
Caminicella	8	0.01%
Eisenbergiella	7	0.01%
Marvinbryantia	7	0.01%
Sphaerochaeta	7	0.01%
unclassified Planctomycetales	7	0.01%
Oligosphaera	7	0.01%
unclassified Bacteroidaceae	6	0.01%
Faecalitalea	6	0.01%

Genus	Number of reads	Relative abundance
Robinsoniella	6	0.01%
Cutibacterium	6	0.01%
Petrimonas	6	0.01%
Anaeroplasma	6	0.01%
Mobilitalea	6	0.01%
Escherichia	6	0.01%
Parvibacter	6	0.01%
Catabacter	5	0.01%
Senegalimassilia	5	0.01%
unclassified Erysipelotrichia	5	0.01%
Natronincola	5	0.01%
Bifidobacterium	5	0.01%
Flavonifractor	5	0.01%
Pediococcus	4	0%
Candidatus Heliomonas	4	0%
Geosporobacter	4	0%
Oceanirhabdus	4	0%
Anaerofilum	4	0%
Dehalobacterium	4	0%
Asteroleplasma	4	0%
Gottschalkia	4	0%
Allisonella	4	0%
Catonella	3	0%
Macellibacteroides	3	0%
Hathewaya	3	0%
Casaltella	3	0%
unclassified Clostridiaceae	3	0%
Desulfosporosinus	3	0%
Bariatricus	3	0%
Mageeibacillus	3	0%
Lactonifactor	3	0%
Fibrobacter	3	0%
Defluviitalea	3	0%
Parasporobacterium	3	0%
unclassified Lactobacillaceae	3	0%
Breznakia	3	0%
Atopobium	2	0%
Oxobacter	2	0%
Howardella	2	0%
Mucispirillum	2	0%
Wautersiella	2	0%
Adlercreutzia	2	0%
unclassified Gammaproteobacteria	2	0%
Anaerosporobacter	2	0%
Anaerococcus	2	0%
Caproiciproducens	2	0%
Cytophaga	2	0%

Genus	Number of reads	Relative abundance
Anaerotruncus	2	0%
Bulleidia	2	0%
Anaerobiospirillum	2	0%
unclassified Betaproteobacteria	2	0%
Clostridioides	2	0%
Caloranaerobacter	2	0%
Garciella	2	0%
Paraeggerthella	2	0%
Syntrophococcus	2	0%
Proteinivorax	2	0%
Gemella	1	0%
Clostridiisalibacter	1	0%
Pseudoramibacter	1	0%
Dethiosulfatibacter	1	0%
Diaminobutyricimonas	1	0%
Dielma	1	0%
Centipeda	1	0%
Tepidibacter	1	0%
unclassified Synergistetes	1	0%
Cryptanaerobacter	1	0%
Alistipes	1	0%
Paenibacillus	1	0%
unclassified Alphaproteobacteria	1	0%
Ercella	1	0%
Pyramidobacter	1	0%
Desnuesiella	1	0%
Kosakonia	1	0%
Sphingobacterium	1	0%
Rummeliibacillus	1	0%
Acholeplasma	1	0%
Pleomorphochaeta	1	0%
Peptoclostridium	1	0%
Sedimentibacter	1	0%
Candidatus Methanoplasma	1	0%
Cellulosibacter	1	0%
Acetoanaerobium	1	0%
Shigella	1	0%
Caldicoprobacter	1	0%
Olavius	1	0%
Paraclostridium	1	0%
Peptostreptococcus	1	0%
Alkaliphilus	1	0%
Proteocatella	1	0%
Larkinella	1	0%
Candidatus Stoquefichus	1	0%
Erythrobacter	1	0%
Aeromicrobium	1	0%

Genus	Number of reads	Relative abundance
Listeria	1	0%
Proteiniborus	1	0%
Acetatifactor	1	0%
Paracoccus	1	0%
Shuttleworthia	1	0%
Kiloniella	1	0%
Rhodoglobus	1	0%
Marinilabilia	1	0%
Candidatus Nardonella	1	0%
Methanobrevibacter	1	0%
Agromyces	1	0%
Hydrogenoanaerobacterium	1	0%
Helcococcus	1	0%
Herbivorax	1	0%
Crassaminicella	1	0%
Desulfomonile	1	0%
Prolixibacter	1	0%
Rhodospirillum	1	0%
Synechococcus	1	0%
Okadaella	1	0%
Alkalibacterium	1	0%
