# Supplementary material for: Combination of Antimicrobial Starters for Feed Fermentation: Influence on Piglet Feces Microbiota and Health and Growth Performance, Including Mycotoxin Biotransformation in vivo
Source: Front Vet Sci. 2020 Oct 16;7:528990. doi: 10.3389/fvets.2020.528990 (PMC7596189; doi:10.3389/fvets.2020.528990)
Supplement: Supplementary File 5 — Control group after experiment species. [file Data_Sheet_5.PDF]

BaseClear Genome Explorer

Species	Number of reads	Relative abundance
Prevotella copri	13326	21.81%
Unclassified	2458	4.02%
Faecalibacterium prausnitzii	2394	3.91%
Terrisporobacter glycolicus	1931	3.16%
Roseburia faecis	1927	3.15%
Prevotella oralis	1824	2.98%
Prevotella histicola	1769	2.89%
Gemmiger formicilis	1584	2.59%
Prevotella brevis	1508	2.46%
Prevotella stercorea	1408	2.3%
Prevotella oris	1261	2.06%
Megasphaera elsdenii	1066	1.74%
Parabacteroides distasonis	976	1.59%
Blautia wexlerae	856	1.4%
Clostridium cellulovorans	840	1.37%
Dialister succinatiphilus	811	1.32%
Alloprevotella rava	714	1.16%
Collinsella aerofaciens	685	1.12%
Romboutsia sedimentorum	626	1.02%
Barnesiella intestinihominis	599	0.98%
Eubacterium rectale	580	0.94%
Flintibacter butyricus	557	0.91%
Butyricicoccus pullicaecorum	539	0.88%
Lactobacillus amylovorus	528	0.86%
Oscillibacter ruminantium	504	0.82%
Enorma massiliensis	465	0.76%
Olsenella umbonata	452	0.74%
Anaerovibrio lipolyticus	400	0.65%
Oscillospira guilliermondii	395	0.64%
Sporobacter termitidis	384	0.62%
Fusicatenibacter saccharivorans	377	0.61%
Prevotella maculosa	361	0.59%
unclassified Barnesiella	360	0.58%
Clostridium celatum	336	0.55%
Eubacterium ramulus	296	0.48%
Eubacterium coprostanoligenes	289	0.47%
Intestinimonas butyriciproducens	287	0.46%
Intestinibacter bartlettii	276	0.45%
Catenibacterium mitsuokai	275	0.45%
Mitsuokella jalaludinii	271	0.44%
Bacteroidales oral	267	0.43%
Prevotella denticola	259	0.42%
Murimonas intestini	256	0.41%
Prevotella salivae	255	0.41%

Species	Number of reads	Relative abundance
Eubacterium hallii	248	0.4%
Prevotella dentalis	246	0.4%
Blautia obeum	246	0.4%
Coprococcus comes	244	0.39%
Ruminococcus faecis	243	0.39%
Ruminococcus flavefaciens	231	0.37%
Paraprevotella clara	218	0.35%
unclassified Prevotella	217	0.35%
Blautia massiliensis	216	0.35%
Prevotella genomosp.	211	0.34%
Prevotella ruminicola	189	0.3%
Enterorhabdus mucosicola	187	0.3%
Phascolarctobacterium succinatutens	169	0.27%
Prevotella loescheii	153	0.25%
Ruminococcus torques	152	0.24%
Ruminococcus bromii	151	0.24%
Eubacterium eligens	145	0.23%
Enterorhabdus caecimuris	141	0.23%
Eubacterium ruminantium	138	0.22%
Olsenella scatoligenes	137	0.22%
Prevotella conceptionensis	133	0.21%
unclassified Lachnospiraceae	133	0.21%
Denitrobacterium detoxificans	132	0.21%
Blautia faecis	131	0.21%
unclassified Bacteroidales	131	0.21%
Fournierella massiliensis	130	0.21%
Anaerotaenia torta	125	0.2%
Ruminococcus callidus	120	0.19%
Dorea longicatena	117	0.19%
Desulfovibrio fairfieldensis	117	0.19%
Butyrivibrio fibrisolvens	117	0.19%
Holdemanella biformis	108	0.17%
Ruminiclostridium thermocellum	104	0.17%
Olsenella uli	103	0.16%
Clostridium aldenense	102	0.16%
Acetivibrio ethanolgignens	100	0.16%
Prevotella dentasini	94	0.15%
Coprococcus catus	90	0.14%
Dorea formicigenerans	87	0.14%
unclassified Tannerella	84	0.13%
Blautia luti	84	0.13%
Coprococcus eutactus	82	0.13%
Roseburia hominis	82	0.13%
Ruminococcus bicirculans	81	0.13%
Lachnospira pectinoschiza	80	0.13%
unclassified Prevotellaceae	79	0.12%
Lactobacillus pontis	78	0.12%

Species	Number of reads	Relative abundance
Eubacterium siraeum	77	0.12%
Intestinimonas massiliensis	76	0.12%
Blautia glucerasea	75	0.12%
cyanobacterium enrichment	72	0.11%
Solobacterium moorei	71	0.11%
Eubacterium desmolans	69	0.11%
Treponema zioleckii	67	0.1%
Clostridium lavalense	67	0.1%
Clostridium leptum	66	0.1%
Mogibacterium diversum	66	0.1%
Oribacterium sinus	66	0.1%
Anaerovorax odorimutans	66	0.1%
Clostridium phoceensis	65	0.1%
Clostridium polysaccharolyticum	65	0.1%
Macellibacteroides fermentans	65	0.1%
Mitsuokella multacida	64	0.1%
Intestinimonas timonensis	63	0.1%
Falcatimonas natans	62	0.1%
Clostridium chartatabidum	61	0.09%
Methanosphaera cuniculi	61	0.09%
Clostridium quinii	60	0.09%
Roseburia inulinivorans	60	0.09%
unclassified Ruminococcaceae	57	0.09%
Treponema berlinense	56	0.09%
Prevotella paludivivens	55	0.09%
Candidatus Dorea	55	0.09%
unclassified Erysipelotrichaceae	55	0.09%
Gracilibacter thermotolerans	54	0.08%
unclassified Clostridium	54	0.08%
Clostridium oroticum	53	0.08%
unclassified Deltaproteobacteria	52	0.08%
Asteroleplasma anaerobium	51	0.08%
Clostridium symbiosum	49	0.08%
Asaccharospora irregularis	49	0.08%
Turicibacter sanguinis	49	0.08%
Anaerobacterium chartisolvens	49	0.08%
Hungatella hathewayi	45	0.07%
Hespellia porcina	45	0.07%
Lutispora thermophila	45	0.07%
Anaerostipes hadrus	44	0.07%
Clostridium amylolyticum	44	0.07%
Natranaerovirga pectinivora	43	0.07%
Prevotella fusca	42	0.06%
Acetanaerobacterium elongatum	42	0.06%
Christensenella minuta	42	0.06%
unclassified Turicibacter	42	0.06%
unclassified Clostridiales	41	0.06%

Species	Number of reads	Relative abundance
Candidatus Soleaferrea	40	0.06%
Erysipelothrix inopinata	40	0.06%
Desulfovibrio piger	39	0.06%
Lactobacillus crispatus	39	0.06%
Clostridium cellobioparum	39	0.06%
Anaerobium acetethylicum	38	0.06%
Eubacterium oxidoreducens	38	0.06%
Collinsella intestinalis	37	0.06%
Slackia isoflavoniconvertens	37	0.06%
Ruthenibacterium lactatiformans	36	0.05%
Sutterella stercoricanis	36	0.05%
Olsenella profusa	36	0.05%
Clostridium fusiformis	35	0.05%
Pseudoflavonifractor capillosus	35	0.05%
Saccharofermentans acetigenes	35	0.05%
Prevotella shahii	35	0.05%
Blautia schinkii	34	0.05%
Clostridium xylanolyticum	34	0.05%
Lactobacillus delbrueckii	34	0.05%
Vallitalea pronyensis	34	0.05%
Prevotella scopos	34	0.05%
Roseburia intestinalis	33	0.05%
Gorbachella massiliensis	33	0.05%
unclassified Rikenella	33	0.05%
Clostridium saccharolyticum	33	0.05%
Anaeromassilibacillus senegalensis	32	0.05%
Clostridium methylpentosum	31	0.05%
Clostridium populeti	31	0.05%
Eubacteriaceae oral	30	0.04%
Ruminococcus albus	30	0.04%
Papillibacter cinnamivorans	29	0.04%
Blautia stercoris	29	0.04%
Holdemania filiformis	29	0.04%
Lactobacillus kitasatonis	28	0.04%
Intestinimonas gabonensis	28	0.04%
Eisenbergiella tayi	27	0.04%
Subdoligranulum variabile	27	0.04%
Prevotella baroniae	27	0.04%
Barnesiella viscericola	26	0.04%
Treponema porcinum	26	0.04%
Clostridium fimetarium	26	0.04%
Parvibacter caecicola	26	0.04%
Prevotella buccalis	26	0.04%
Acetivibrio cellulolyticus	25	0.04%
Clostridium disporicum	25	0.04%
Peptococcus simiae	25	0.04%
Lactobacillus panis	25	0.04%

Species	Number of reads	Relative abundance
Lactobacillus jensenii	25	0.04%
Selenomonas ruminantium	25	0.04%
Ruminococcus gnavus	24	0.03%
Prevotella bivia	24	0.03%
Clostridium asparagiforme	24	0.03%
Paeniclostridium sordellii	24	0.03%
Oligosphaera ethanolica	23	0.03%
Clostridium sartagoforme	23	0.03%
Escherichia coli	23	0.03%
unclassified Paludibacter	23	0.03%
Eubacterium tenue	23	0.03%
Agathobacter ruminis	23	0.03%
Clostridium clostridioforme	23	0.03%
Eubacterium rangiferina	22	0.03%
unclassified Clostridia	22	0.03%
Porphyromonas catoniae	22	0.03%
Bacteroides pectinophilus	22	0.03%
Prevotella melaninogenica	22	0.03%
Clostridium intestinale	21	0.03%
Corynebacterium provencense	21	0.03%
Marvinbryantia formatexigens	21	0.03%
Prevotella timonensis	21	0.03%
Clostridium aminobutyricum	20	0.03%
unclassified Mollicutes	20	0.03%
Ethanoligenens harbinense	20	0.03%
unclassified Anaerovibrio	20	0.03%
Oscillibacter valericigenes	19	0.03%
Clostridium longisporum	19	0.03%
unclassified Bacteroides	19	0.03%
Staphylococcus epidermidis	19	0.03%
Selenomonas sputigena	19	0.03%
Eubacterium cellulosolvens	18	0.02%
Clostridium hveragerdense	18	0.02%
Allisonella histaminiformans	18	0.02%
Abyssivirga alkaniphila	18	0.02%
Bacteroidales genomosp.	18	0.02%
Clostridium cellulolyticum	18	0.02%
Clostridium tertium	17	0.02%
Treponema succinifaciens	17	0.02%
Clostridium chauvoei	17	0.02%
Eubacterium contortum	17	0.02%
Prevotella buccae	17	0.02%
unclassified Planctomycetales	17	0.02%
Butyrivibrio crossotus	17	0.02%
Clostridium cadaveris	16	0.02%
Collinsella massiliensis	16	0.02%
Flavonifractor plautii	16	0.02%

Species	Number of reads	Relative abundance
Robinsoniella peoriensis	16	0.02%
Lactobacillus frumenti	16	0.02%
Paraeggerthella hongkongensis	16	0.02%
Hallella seregens	16	0.02%
unclassified Dialister	15	0.02%
Coprobacillus cateniformis	15	0.02%
Collinsella stercoris	15	0.02%
Clostridium papyrosolvens	15	0.02%
Lachnoanaerobaculum umeaense	15	0.02%
Clostridium hiranonis	15	0.02%
Bacteroides caecicola	14	0.02%
Herbinix luporum	14	0.02%
Lactobacillus secaliphilus	14	0.02%
Eubacterium plexicaudatum	14	0.02%
Prevotella saccharolytica	14	0.02%
Caminicella sporogenes	13	0.02%
Fibrobacter intestinalis	13	0.02%
Lactobacillus helveticus	13	0.02%
Selenomonas bovis	13	0.02%
Lactobacillus rogosae	13	0.02%
unclassified Oscillospira	13	0.02%
Lachnoanaerobaculum saburreum	13	0.02%
Eubacterium ventriosum	13	0.02%
Clostridioides difficile	13	0.02%
Elbe River	13	0.02%
Clostridium clariflavum	13	0.02%
Natronincola histidinovorans	13	0.02%
Desulfovibrio desulfuricans	12	0.01%
Anaerostipes butyraticus	12	0.01%
unclassified Porphyromonadaceae	12	0.01%
Clostridium bovipellis	12	0.01%
Clostridium sphenoides	12	0.01%
Geosporobacter ferrireducens	12	0.01%
Bacteroides galacturonicus	12	0.01%
Prevotella enoeca	12	0.01%
Parabacteroides chinchillae	12	0.01%
Mobilitalea sibirica	12	0.01%
Clostridium aerotolerans	11	0.01%
Ruminococcus champanellensis	11	0.01%
Parasutterella secunda	11	0.01%
Ruminococcus lactaris	11	0.01%
unclassified Alloprevotella	11	0.01%
Alloprevotella tannerae	11	0.01%
unclassified Olsenella	11	0.01%
Eisenbergiella massiliensis	11	0.01%
Bacteroides stercoris	11	0.01%
Prevotella jejuni	11	0.01%

Species	Number of reads	Relative abundance
Acidaminobacter hydrogenoformans	11	0.01%
Bacteroides timonensis	10	0.01%
Clostridium hungatei	10	0.01%
Adlercreutzia equolifaciens	10	0.01%
Prevotella nanceiensis	10	0.01%
unclassified Wautersiella	9	0.01%
Erysipelothrix rhusiopathiae	9	0.01%
Clostridium indolis	9	0.01%
unclassified Acetivibrio	9	0.01%
Campylobacter lanienae	9	0.01%
Lactobacillus reuteri	9	0.01%
Clostridium neopropionicum	9	0.01%
unclassified Faecalibacterium	9	0.01%
Lachnoanaerobaculum cf.	9	0.01%
unclassified Eubacterium	9	0.01%
Anaerocolumna cellulosilytica	9	0.01%
Prevotella corporis	9	0.01%
Parasutterella excrementihominis	9	0.01%
Bacteroides heparinolyticus	9	0.01%
Sphaerochaeta coccoides	8	0.01%
Lactonifactor longoviformis	8	0.01%
Cutibacterium acnes	8	0.01%
Candidatus Heliomonas	8	0.01%
Brassicibacter thermophilus	8	0.01%
Staphylococcus capitis	8	0.01%
Dialister propionicifaciens	8	0.01%
Blautia producta	8	0.01%
unclassified Oscillibacter	7	0.01%
Bacteroides clarus	7	0.01%
Prevotella bryantii	7	0.01%
Prevotella micans	7	0.01%
Clostridium lactatifermentans	7	0.01%
Paraclostridium benzoelyticum	7	0.01%
Lachnospira multipara	7	0.01%
actinobacterium enrichment	7	0.01%
Parasporobacterium paucivorans	7	0.01%
Bacteroides intestinalis	7	0.01%
Candidatus Stoquefichus	7	0.01%
Prevotella marshii	7	0.01%
Terrisporobacter mayombei	7	0.01%
methanogenic archaeon	7	0.01%
unclassified Treponema	7	0.01%
Lactobacillus coleohominis	7	0.01%
Lachnobacterium bovis	7	0.01%
Bacteroides uniformis	6	0%
Bacteroides cellulosilyticus	6	0%
Clostridium glycyrrhizinilyticum	6	0%

Species	Number of reads	Relative abundance
Eubacterium infirmum	6	0%
unclassified Bulleidia	6	0%
Atopobium vaginae	6	0%
Candidatus Treponema	6	0%
Clostridium hylemonae	6	0%
Clostridium aurantibutyricum	6	0%
Lactobacillus acidophilus	6	0%
Bariatricus massiliensis	6	0%
Porphyromonas pasteri	6	0%
Clostridium islandicum	6	0%
Enorma timonensis	6	0%
unclassified Lactobacillus	6	0%
unclassified Cryptanaerobacter	6	0%
Clostridium butyricum	6	0%
unclassified Ruminococcus	6	0%
Breznakia pachnodae	5	0%
Paeniclostridium ghonii	5	0%
Bifidobacterium pseudolongum	5	0%
Clostridium scindens	5	0%
Bacteroides salanitronis	5	0%
Clostridium celerecrescens	5	0%
unclassified Bacillus	5	0%
Pseudobutyrivibrio ruminis	5	0%
Hespellia stercorisuis	5	0%
Prevotella veroralis	5	0%
Clostridium straminisolvens	5	0%
Clostridium botulinum	5	0%
Paraclostridium bifermentans	5	0%
Bacteroides barnesiae	5	0%
Caloramator fervidus	5	0%
Dielma fastidiosa	5	0%
Terrisporobacter petrolearius	5	0%
Stomatobaculum longum	5	0%
Anaerosporobacter mobilis	5	0%
Clostridium purinilyticum	5	0%
Caproiciproducens galactitolivorans	5	0%
unclassified Subdoligranulum	5	0%
Prevotella albensis	4	0%
Megasphaera hominis	4	0%
Ruminococcus gauvreauii	4	0%
Pleomorphochaeta multiformis	4	0%
unclassified Bacteroidaceae	4	0%
Sphaerochaeta pleomorpha	4	0%
Porphyromonas pogonae	4	0%
Anaerofilum pentosovorans	4	0%
Parabacteroides merdae	4	0%
Bacteroides caecigallinarum	4	0%

Species	Number of reads	Relative abundance
Bacteroides helcogenes	4	0%
Anaeroplasma abactoclasticum	4	0%
Bacteroides nordii	4	0%
Syntrophococcus sucromutans	4	0%
Bacteroides zoogleoformans	4	0%
unclassified Porphyromonas	4	0%
Senegalimassilia anaerobia	4	0%
Dehalobacterium formicoaceticum	4	0%
Geosporobacter subterraneus	4	0%
Slackia exigua	4	0%
Drancourtella massiliensis	4	0%
Sutterella wadsworthensis	4	0%
Desulfosporosinus orientis	4	0%
Clostridium isatidis	4	0%
unclassified Betaproteobacteria	4	0%
Clostridium putrefaciens	4	0%
Acetatifactor muris	4	0%
Eubacterium sulci	3	0%
Anaerocolumna aminovalerica	3	0%
Catabacter hongkongensis	3	0%
Bacteroides faecichinchillae	3	0%
Desulfotomaculum halophilum	3	0%
Eubacterium dolichum	3	0%
Clostridium sulfidigenes	3	0%
unclassified Clostridiales Family XIII. Incertae Sedis	3	0%
Peptococcus niger	3	0%
Prevotella amnii	3	0%
Clostridium colicanis	3	0%
Defluviitalea raffinosedens	3	0%
Clostridium cellulosi	3	0%
Thermotalea metallivorans	3	0%
Clostridium moniliforme	3	0%
Clostridium diolis	3	0%
Bacillus panaciterrae	3	0%
Gordonibacter urolithinfaciens	3	0%
Clostridium bolteae	3	0%
Pseudobutyrivibrio xylanivorans	3	0%
Prevotella oulorum	3	0%
Anaerotruncus colihominis	3	0%
unclassified Peptococcaceae	3	0%
Mucispirillum schaedleri	3	0%
Blautia coccoides	3	0%
Proteocatella sphenisci	3	0%
Erysipelatoclostridium ramosum	3	0%
Megasphaera paucivorans	3	0%
Eubacterium saphenum	3	0%
Acetoanaerobium sticklandii	3	0%

Species	Number of reads	Relative abundance
Blautia hydrogenotrophica	3	0%
Prevotella multiformis	3	0%
Eubacterium xylanophilum	3	0%
unclassified Spirochaetia	3	0%
Parabacteroides goldsteinii	3	0%
Helicobacter rodentium	3	0%
Bacteroidales str.	3	0%
Lachnoclostridium phytofermentans	3	0%
Succinivibrio dextrinosolvens	3	0%
Casaltella massiliensis	2	0%
unclassified Bifidobacterium	2	0%
Anaerobiospirillum succiniciproducens	2	0%
Clostridium termitidis	2	0%
Oceanirhabdus sediminicola	2	0%
Staphylococcus haemolyticus	2	0%
Anaerostipes rhamnosivorans	2	0%
Clostridium stercorarium	2	0%
Staphylococcus caprae	2	0%
Oxobacter pfennigii	2	0%
Listeria monocytogenes	2	0%
unclassified Anaerovorax	2	0%
Staphylococcus chromogenes	2	0%
Treponema brennaborense	2	0%
Clostridium aminophilum	2	0%
unclassified Victivallaceae	2	0%
Clostridium amygdalinum	2	0%
Desulfotomaculum nigrificans	2	0%
Prevotella aff.	2	0%
Anaerococcus provenciensis	2	0%
Acetitomaculum ruminis	2	0%
Corynebacterium xerosis	2	0%
unclassified Papillibacter	2	0%
alpha proteobacterium	2	0%
unclassified Clostridiaceae	2	0%
Acidaminococcus fermentans	2	0%
Bulleidia extructa	2	0%
Bacillus pumilus	2	0%
Oribacterium parvum	2	0%
Lactobacillus mucosae	2	0%
Atopobium rimae	2	0%
Treponema parvum	2	0%
unclassified Desulfovibrio	2	0%
Acetoanaerobium pronyense	2	0%
unclassified Sporobacter	2	0%
Slackia piriformis	2	0%
Dysgonomonas termitidis	2	0%
unclassified Erysipelotrichia	2	0%

Species	Number of reads	Relative abundance
Oxalobacter formigenes	2	0%
Collinsella tanakaei	2	0%
Lactobacillus johnsonii	2	0%
Anaerocolumna xylanovorans	2	0%
Clostridium cavendishii	2	0%
Eubacterium pyruvativorans	2	0%
unclassified Catonella	2	0%
Sutterella parvirubra	2	0%
unclassified Oribacterium	2	0%
beta proteobacterium	2	0%
Lactobacillus amylolyticus	2	0%
unclassified Veillonellaceae	2	0%
Moorella humiferrea	2	0%
Desulfosporosinus acidiphilus	2	0%
Clostridium carnis	2	0%
Haloplasma contractile	2	0%
Pyramidobacter piscolens	1	0%
unclassified Schlegelella	1	0%
Cellulosibacter alkalithermophilus	1	0%
Dongia mobilis	1	0%
Fastidiosipila sanguinis	1	0%
Desulfitobacterium chlororespirans	1	0%
Clostridium septicum	1	0%
Acinetobacter baumannii	1	0%
Eggerthella lenta	1	0%
Actinomyces provencensis	1	0%
unclassified Paenibacillus	1	0%
Tyzzerella nexilis	1	0%
Centipeda periodontii	1	0%
Chelativorans composti	1	0%
unclassified Coriobacteriaceae	1	0%
Bacteroides stercorirosoris	1	0%
Gardnerella vaginalis	1	0%
Peptoclostridium litorale	1	0%
Bacteroides gallinaceum	1	0%
Peptoclostridium acidaminophilum	1	0%
Methyloterrigena soli	1	0%
metal-contaminated soil	1	0%
Bacteroides vulgatus	1	0%
Brevibacillus fulvus	1	0%
Asaccharobacter celatus	1	0%
unclassified Dorea	1	0%
Bacteroides oleiciplenus	1	0%
Clostridium tyrobutyricum	1	0%
Clostridium sufflavum	1	0%
Bifidobacterium choerinum	1	0%
Pelobacter carbinolicus	1	0%

Species	Number of reads	Relative abundance
unclassified Actinobacteria	1	0%
unclassified Propionibacterium	1	0%
Prevotella intermedia	1	0%
Bittarella massiliensis	1	0%
Clostridium paraputrificum	1	0%
unclassified Sphingobium	1	0%
unclassified Collinsella	1	0%
Lactobacillus psittaci	1	0%
unclassified Proteus	1	0%
Anaerorhabdus furcosa	1	0%
Porphyromonas crevioricanis	1	0%
Faecalicoccus acidiformans	1	0%
unclassified Thiobaca	1	0%
Defluviitalea phaphyphila	1	0%
Blautia hansenii	1	0%
Sphaerochaeta globosa	1	0%
Kluyvera georgiana	1	0%
Prevotella nigrescens	1	0%
unclassified Epsilonproteobacteria	1	0%
unclassified Pseudobutyrivibrio	1	0%
Bacteroides coprophilus	1	0%
unclassified Lactobacillaceae	1	0%
Bacteroides luti	1	0%
Clostridium alkalicellulosi	1	0%
Bifidobacterium longum	1	0%
Eubacterium fissicatena	1	0%
Caloranaerobacter azorensis	1	0%
Cryocola antiquus	1	0%
Bacteroides xylanolyticus	1	0%
Porphyromonas canoris	1	0%
unclassified Adlercreutzia	1	0%
Anaeroplasma bactoclasticum	1	0%
Paracoccus marinus	1	0%
Lactobacillus brevis	1	0%
Lawsonella clevelandensis	1	0%
Dethiosulfatibacter aminovorans	1	0%
Romboutsia lituseburensis	1	0%
Clostridium viride	1	0%
Prevotella bergensis	1	0%
Staphylococcus cohnii	1	0%
unclassified Streptococcaceae	1	0%
Sphingobium jiangsuense	1	0%
Proteinivorax tanatarense	1	0%
Solitalea koreensis	1	0%
Helicobacter ganmani	1	0%
Herbinix hemicellulosilytica	1	0%
Hungatella effluvii	1	0%

Species	Number of reads	Relative abundance
Prevotella oryzae	1	0%
Bacillus hwajinpoensis	1	0%
Eubacterium minutum	1	0%
Defluviitalea saccharophila	1	0%
Bacteroides graminisolvens	1	0%
unclassified Gordonibacter	1	0%
Clostridium josui	1	0%
Vallitalea guaymasensis	1	0%
Bifidobacterium saguini	1	0%
unclassified Asteroleplasma	1	0%
Mogibacterium timidum	1	0%
Tannerella forsythia	1	0%
Bacteroides salyersiae	1	0%
Bacteroides neonati	1	0%
unclassified Peptococcus	1	0%
Selenomonas noxia	1	0%
Cellulosilyticum ruminicola	1	0%
Propionibacterium namnetense	1	0%
Clostridium propionicum	1	0%
Clostridium baratii	1	0%
Caloramator viterbiensis	1	0%
Propionispira paucivorans	1	0%
Clostridium ihumii	1	0%
Lactobacillus fermentum	1	0%
unclassified Petrimonas	1	0%
unclassified Eubacteriaceae	1	0%
Alkaliphilus metalliredigens	1	0%
Selenomonas genomosp.	1	0%
Acidaminococcus intestini	1	0%
Kandleria vitulina	1	0%
Desulfotomaculum guttoideum	1	0%
Cutibacterium granulosum	1	0%
Lactobacillus gallinarum	1	0%
Acholeplasma parvum	1	0%
Holdemania massiliensis	1	0%
Lactobacillus hamsteri	1	0%
unclassified Catenuloplanes	1	0%
Prevotella multisaccharivorax	1	0%
Fucophilus fucoidanolyticus	1	0%
Paraburkholderia andropogonis	1	0%
Tepidibacter mesophilus	1	0%
Selenomonas lacticifex	1	0%
unclassified Kopriimonas	1	0%
unclassified Magnetococcus	1	0%
Bacteroides ovatus	1	0%
Garciella nitratireducens	1	0%
Clostridium taeniosporum	1	0%

Species	Number of reads	Relative abundance
Sphingoaurantiacus polygranulatus	1	0%
Chroococcales cyanobacterium	1	0%
Desulfotomaculum thermosapovorans	1	0%
Alistipes massiliensis	1	0%
unclassified Melghirimyces	1	0%
Clostridium cocleatum	1	0%
Clostridium neonatale	1	0%
Campylobacter jejuni	1	0%
Nesterenkonia aethiopica	1	0%
Sellimonas intestinalis	1	0%
Lactobacillus parabuchneri	1	0%
Clostridium beijerinckii	1	0%
Staphylococcus saccharolyticus	1	0%
Bacteroides paurosaccharolyticus	1	0%
unclassified Streptacidiphilus	1	0%
Lactobacillus animalis	1	0%
Catonella morbi	1	0%
Staphylococcus kloosii	1	0%
marine alpha	1	0%
unclassified Coprococcus	1	0%
Gottschalkia acidurici	1	0%
Alistipes putredinis	1	0%
Bifidobacterium animalis	1	0%
unclassified Erysipelothrix	1	0%
Lactobacillus suebicus	1	0%
unclassified Geobacter	1	0%
Eubacterium limosum	1	0%
Prevotella disiens	1	0%
Tepidimicrobium xylanilyticum	1	0%
Romboutsia ilealis	1	0%
Candidatus Izimaplasma	1	0%
Bacteroides dorei	1	0%
Izhakiella capsodis	1	0%
Streptococcus minor	1	0%
Marinithermofilum abyssi	1	0%
Slackia equolifaciens	1	0%
Schwartzia succinivorans	1	0%
Candidatus Methanomethylophilus	1	0%
Fenollaria massiliensis	1	0%
Shigella dysenteriae	1	0%
Lactobacillus vaginalis	1	0%
Moraxella lacunata	1	0%
Kosakonia sacchari	1	0%
Aeromonas hydrophila	1	0%
Clostridium fallax	1	0%
Parabacteroides gordonii	1	0%
Snodgrassella alvi	1	0%

Species	Number of reads	Relative abundance
Streptococcus azizii	1	0%
unclassified Desulfomicrobiaceae	1	0%
Desulfotomaculum hydrothermale	1	0%
Desulfotomaculum gibsoniae	1	0%
Clostridium hydrogeniformans	1	0%
Pediococcus acidilactici	1	0%
Actinocorallia cavernae	1	0%
Cryptobacterium curtum	1	0%
Shuttleworthia satelles	1	0%
unclassified Gammaproteobacteria	1	0%
Moorella stamsii	1	0%
Thermanaerovibrio velox	1	0%
Alkalibacter saccharofermentans	1	0%
