# Supplementary material for: Combination of Antimicrobial Starters for Feed Fermentation: Influence on Piglet Feces Microbiota and Health and Growth Performance, Including Mycotoxin Biotransformation in vivo
Source: Front Vet Sci. 2020 Oct 16;7:528990. doi: 10.3389/fvets.2020.528990 (PMC7596189; doi:10.3389/fvets.2020.528990)
Supplement: Supplementary File 6 — Experimental group after experiment species. [file Data_Sheet_6.PDF]

BaseClear Genome Explorer

Species	Number of reads	Relative abundance
Lactobacillus amylovorus	9389	19.39%
Prevotella copri	9001	18.59%
Prevotella stercorea	2247	4.64%
Clostridium cellulovorans	1977	4.08%
Prevotella oralis	1665	3.43%
Eubacterium rectale	1351	2.79%
Unclassified	1018	2.1%
Terrisporobacter glycolicus	926	1.91%
Lactobacillus reuteri	899	1.85%
Faecalibacterium prausnitzii	839	1.73%
Prevotella oris	790	1.63%
Megasphaera elsdenii	736	1.52%
Alloprevotella rava	722	1.49%
Sporobacter termitidis	583	1.2%
Gemmiger formicilis	569	1.17%
Prevotella brevis	518	1.06%
Anaerovibrio lipolyticus	467	0.96%
Butyricicoccus pullicaecorum	459	0.94%
Streptococcus lutetiensis	444	0.91%
Roseburia faecis	414	0.85%
Prevotella dentalis	347	0.71%
Barnesiella intestinihominis	305	0.62%
Paraprevotella clara	252	0.52%
Streptococcus gallolyticus	242	0.49%
Flintibacter butyricus	237	0.48%
Eubacterium coprostanoligenes	221	0.45%
Bacteroidales oral	221	0.45%
Phascolarctobacterium succinatutens	214	0.44%
Blautia wexlerae	210	0.43%
Intestinibacter bartlettii	191	0.39%
Parabacteroides goldsteinii	191	0.39%
Clostridium celatum	185	0.38%
Prevotella genomosp.	183	0.37%
Lactobacillus johnsonii	167	0.34%
Butyrivibrio fibrisolvens	166	0.34%
Prevotella histicola	158	0.32%
Lactobacillus jensenii	155	0.32%
Intestinimonas butyriciproducens	152	0.31%
Clostridium quinii	151	0.31%
unclassified Barnesiella	150	0.3%
Lactobacillus crispatus	148	0.3%
Fusicatenibacter saccharivorans	145	0.29%
Lactobacillus delbrueckii	143	0.29%
Ruminiclostridium thermocellum	139	0.28%

Species	Number of reads	Relative abundance
unclassified Prevotella	139	0.28%
Catenibacterium mitsuokai	136	0.28%
Parabacteroides distasonis	130	0.26%
Lactobacillus pontis	125	0.25%
Oscillibacter ruminantium	119	0.24%
Prevotella conceptionensis	119	0.24%
Eubacterium ruminantium	114	0.23%
Prevotella paludivivens	106	0.21%
Holdemanella biformis	103	0.21%
Ruminococcus bicirculans	102	0.21%
Oscillospira guilliermondii	102	0.21%
Lactobacillus kitasatonis	102	0.21%
Romboutsia sedimentorum	101	0.2%
Ruminococcus flavefaciens	98	0.2%
Prevotella salivae	98	0.2%
Blautia obeum	93	0.19%
Coprococcus catus	88	0.18%
unclassified Bacteroidales	87	0.17%
Blautia producta	85	0.17%
Prevotella ruminicola	85	0.17%
Lactobacillus helveticus	85	0.17%
Eubacterium hallii	83	0.17%
Eubacterium ramulus	82	0.16%
Ruminococcus faecis	81	0.16%
Ruminococcus torques	81	0.16%
Ruminococcus bromii	79	0.16%
Mitsuokella multacida	77	0.15%
Blautia massiliensis	72	0.14%
Eubacterium siraeum	71	0.14%
Prevotella maculosa	70	0.14%
unclassified Turicibacter	70	0.14%
Clostridium bovipellis	70	0.14%
Coprococcus comes	68	0.14%
Murimonas intestini	68	0.14%
Prevotella loescheii	67	0.13%
unclassified Lachnospiraceae	65	0.13%
Roseburia hominis	63	0.13%
Collinsella aerofaciens	62	0.12%
Clostridium chartatabidum	62	0.12%
Anaerotaenia torta	57	0.11%
Roseburia inulinivorans	56	0.11%
Clostridium amylolyticum	55	0.11%
Clostridium aldenense	54	0.11%
Solobacterium moorei	53	0.1%
Candidatus Soleaferrea	51	0.1%
unclassified Tannerella	50	0.1%
Lachnospira pectinoschiza	48	0.09%

Species	Number of reads	Relative abundance
Mitsuokella jalaludinii	48	0.09%
Vallitalea pronyensis	48	0.09%
cyanobacterium enrichment	47	0.09%
Streptococcus danieliae	46	0.09%
Fournierella massiliensis	46	0.09%
Dialister succinatiphilus	46	0.09%
Dorea longicatena	45	0.09%
Christensenella minuta	44	0.09%
Agathobacter ruminis	44	0.09%
Olsenella scatoligenes	43	0.08%
Clostridium polysaccharolyticum	42	0.08%
Intestinimonas timonensis	41	0.08%
Dorea formicigenerans	41	0.08%
Roseburia intestinalis	41	0.08%
Lactobacillus acidophilus	41	0.08%
unclassified Clostridium	40	0.08%
Brassicibacter thermophilus	40	0.08%
Sutterella stercoricanis	38	0.07%
Lactobacillus mucosae	38	0.07%
Mogibacterium diversum	37	0.07%
Streptococcus equinus	37	0.07%
Blautia stercoris	36	0.07%
Bacteroides barnesiae	35	0.07%
Clostridium phoceensis	35	0.07%
Clostridium cellobioparum	34	0.07%
Clostridium lavalense	34	0.07%
unclassified Erysipelotrichaceae	34	0.07%
Eubacterium eligens	34	0.07%
Succinivibrio dextrinosolvens	33	0.06%
Clostridium methylpentosum	33	0.06%
Acetivibrio ethanolgignens	33	0.06%
Gracilibacter thermotolerans	32	0.06%
Eubacterium desmolans	32	0.06%
Porphyromonas catoniae	32	0.06%
Anaerobacterium chartisolvens	32	0.06%
Desulfovibrio piger	32	0.06%
Hungatella hathewayi	31	0.06%
Anaerostipes hadrus	31	0.06%
Clostridium leptum	31	0.06%
Prevotella denticola	29	0.05%
Oribacterium sinus	29	0.05%
Blautia luti	29	0.05%
unclassified Paludibacter	29	0.05%
unclassified Ruminococcaceae	28	0.05%
Bacteroidales genomosp.	27	0.05%
Clostridium disporicum	27	0.05%
unclassified Prevotellaceae	27	0.05%

Species	Number of reads	Relative abundance
Acetanaerobacterium elongatum	26	0.05%
Prevotella shahii	26	0.05%
unclassified Mollicutes	26	0.05%
Denitrobacterium detoxificans	26	0.05%
Parabacteroides chinchillae	26	0.05%
Clostridium cadaveris	26	0.05%
Methanosphaera cuniculi	26	0.05%
Falcatimonas natans	25	0.05%
Olsenella umbonata	25	0.05%
Acidaminobacter hydrogenoformans	25	0.05%
Prevotella bryantii	25	0.05%
Blautia glucerasea	24	0.04%
Anaerocolumna cellulosilytica	24	0.04%
Intestinimonas massiliensis	24	0.04%
Clostridium cellulolyticum	24	0.04%
Barnesiella viscericola	24	0.04%
Coprococcus eutactus	23	0.04%
Lactococcus lactis	23	0.04%
Prevotella buccae	23	0.04%
Asaccharospora irregularis	23	0.04%
Prevotella jejuni	23	0.04%
Blautia faecis	22	0.04%
Turicibacter sanguinis	22	0.04%
Anaerovorax odorimutans	21	0.04%
Lactobacillus amylolyticus	21	0.04%
Erysipelothrix inopinata	21	0.04%
Papillibacter cinnamivorans	21	0.04%
Eubacteriaceae oral	21	0.04%
Clostridium sartagoforme	21	0.04%
Clostridium saccharolyticum	21	0.04%
Parabacteroides johnsonii	20	0.04%
Desulfovibrio fairfieldensis	20	0.04%
Bacteroides pectinophilus	20	0.04%
Prevotella enoeca	20	0.04%
Natranaerovirga pectinivora	20	0.04%
Peptococcus simiae	20	0.04%
Parabacteroides merdae	20	0.04%
Saccharofermentans acetigenes	19	0.03%
Bacteroides coprocola	19	0.03%
Clostridium xylanolyticum	19	0.03%
unclassified Deltaproteobacteria	19	0.03%
Hallella seregens	19	0.03%
Clostridium populeti	19	0.03%
Lactobacillus gasseri	18	0.03%
Prevotella bivia	18	0.03%
Clostridium chauvoei	18	0.03%
Caloramator fervidus	18	0.03%

Species	Number of reads	Relative abundance
Butyrivibrio crossotus	18	0.03%
Prevotella timonensis	18	0.03%
Parasutterella secunda	17	0.03%
Anaerostipes butyraticus	17	0.03%
Clostridium fimetarium	17	0.03%
Enorma massiliensis	17	0.03%
Bacteroides intestinalis	16	0.03%
Blautia schinkii	16	0.03%
Elbe River	16	0.03%
Clostridium oroticum	16	0.03%
Clostridium tarantellae	16	0.03%
Clostridium butyricum	16	0.03%
Lactobacillus rogosae	16	0.03%
Hespellia porcina	16	0.03%
unclassified Porphyromonadaceae	15	0.03%
unclassified Streptococcaceae	15	0.03%
Lactobacillus hamsteri	15	0.03%
Lactobacillus frumenti	15	0.03%
Herbinix luporum	15	0.03%
Holdemania filiformis	15	0.03%
Clostridium aminobutyricum	15	0.03%
Clostridium longisporum	15	0.03%
Ruminococcus champanellensis	15	0.03%
Clostridium bornimense	14	0.02%
Bacteroides salanitronis	14	0.02%
Staphylococcus epidermidis	14	0.02%
Prevotella saccharolytica	14	0.02%
Eubacterium oxidoreducens	14	0.02%
Oscillibacter valericigenes	14	0.02%
Bacteroides helcogenes	14	0.02%
Ruthenibacterium lactatiformans	14	0.02%
unclassified Clostridiales	14	0.02%
Lactobacillus vaginalis	14	0.02%
Eubacterium rangiferina	13	0.02%
unclassified Rikenella	13	0.02%
Streptococcus macedonicus	13	0.02%
Candidatus Dorea	13	0.02%
Clostridium hveragerdense	13	0.02%
Ruminococcus gnavus	13	0.02%
Anaerobium acetethylicum	13	0.02%
Clostridium colicanis	13	0.02%
Ruminococcus albus	12	0.02%
Clostridium hiranonis	12	0.02%
Prevotella albensis	12	0.02%
Clostridium asparagiforme	12	0.02%
unclassified Bacteroides	12	0.02%
Selenomonas ruminantium	12	0.02%

Species	Number of reads	Relative abundance
Clostridium symbiosum	12	0.02%
Ruminococcus lactaris	12	0.02%
Clostridium tertium	12	0.02%
Acetivibrio cellulolyticus	12	0.02%
Intestinimonas gabonensis	12	0.02%
Prevotella scopos	12	0.02%
Lactobacillus hominis	12	0.02%
Desulfotomaculum guttoideum	12	0.02%
unclassified Anaerovibrio	12	0.02%
Ruminococcus callidus	12	0.02%
Bacteroides uniformis	11	0.02%
Clostridium intestinale	11	0.02%
Bacteroides caecigallinarum	11	0.02%
unclassified Clostridia	11	0.02%
Lactobacillus panis	11	0.02%
Corynebacterium provencense	11	0.02%
Eubacterium xylanophilum	11	0.02%
Faecalicoccus acidiformans	11	0.02%
Treponema porcinum	11	0.02%
Clostridium clariflavum	10	0.02%
Eubacterium tenue	10	0.02%
Treponema berlinense	10	0.02%
Paludibacter propionicigenes	10	0.02%
Ethanoligenens harbinense	10	0.02%
methanogenic archaeon	10	0.02%
unclassified Alloprevotella	10	0.02%
Streptococcus porcorum	10	0.02%
Enterorhabdus mucosicola	10	0.02%
Propionispira paucivorans	10	0.02%
Gorbachella massiliensis	10	0.02%
Prevotella buccalis	9	0.01%
Clostridium clostridioforme	9	0.01%
Abyssivirga alkaniphila	9	0.01%
Megasphaera hominis	9	0.01%
Clostridium sulfidigenes	9	0.01%
Acidaminococcus fermentans	9	0.01%
unclassified Acetivibrio	9	0.01%
Prevotella baroniae	9	0.01%
Lachnoanaerobaculum umeaense	9	0.01%
Pseudoflavonifractor capillosus	9	0.01%
Bacteroides stercoris	9	0.01%
Coprobacillus cateniformis	9	0.01%
Prevotella dentasini	9	0.01%
Clostridium fusiformis	9	0.01%
Caminicella sporogenes	8	0.01%
Propionispira arcuata	8	0.01%
Campylobacter lanienae	8	0.01%

Species	Number of reads	Relative abundance
Anaeromassilibacillus senegalensis	8	0.01%
Pseudobutyrivibrio ruminis	8	0.01%
Subdoligranulum variabile	8	0.01%
Prevotella micans	8	0.01%
Prevotella melaninogenica	8	0.01%
Lutispora thermophila	8	0.01%
Clostridium aurantibutyricum	8	0.01%
Clostridium sphenoides	8	0.01%
unclassified Planctomycetales	7	0.01%
Oligosphaera ethanolica	7	0.01%
Streptococcus equi	7	0.01%
Alloprevotella tannerae	7	0.01%
Marvinbryantia formatexigens	7	0.01%
Clostridium celerecrescens	7	0.01%
Helicobacter canadensis	7	0.01%
Lactobacillus psittaci	7	0.01%
Eubacterium sulci	7	0.01%
Bacteroides cellulosilyticus	7	0.01%
Desulfotomaculum tongense	6	0.01%
Clostridium lactatifermentans	6	0.01%
Prevotella amnii	6	0.01%
unclassified Petrimonas	6	0.01%
Paeniclostridium sordellii	6	0.01%
unclassified Ruminococcus	6	0.01%
Eisenbergiella tayi	6	0.01%
unclassified Lactobacillus	6	0.01%
Mobilitalea sibirica	6	0.01%
Clostridium hylemonae	6	0.01%
Clostridium scindens	6	0.01%
Parvibacter caecicola	6	0.01%
Desulfovibrio desulfuricans	6	0.01%
Robinsoniella peoriensis	6	0.01%
Escherichia coli	6	0.01%
Clostridium isatidis	6	0.01%
Clostridium aerotolerans	6	0.01%
Olsenella profusa	6	0.01%
unclassified Eubacterium	6	0.01%
unclassified Bacillus	6	0.01%
unclassified Bacteroidaceae	6	0.01%
Clostridium moniliforme	6	0.01%
Faecalitalea cylindroides	6	0.01%
Prevotella marshii	6	0.01%
Selenomonas bovis	5	0.01%
Natronincola histidinovorans	5	0.01%
Catabacter hongkongensis	5	0.01%
Clostridium glycyrrhizinilyticum	5	0.01%
Lactobacillus antri	5	0.01%

Species	Number of reads	Relative abundance
Olsenella uli	5	0.01%
Prevotella nanceiensis	5	0.01%
unclassified Sporobacter	5	0.01%
Bacteroides plebeius	5	0.01%
Slackia isoflavoniconvertens	5	0.01%
Flavonifractor plautii	5	0.01%
unclassified Porphyromonas	5	0.01%
Parabacteroides gordonii	5	0.01%
Cutibacterium acnes	5	0.01%
Anaeroplasma bactoclasticum	5	0.01%
Senegalimassilia anaerobia	5	0.01%
unclassified Enterococcus	5	0.01%
Anaerocolumna xylanovorans	5	0.01%
Porphyromonas pogonae	5	0.01%
Treponema brennaborense	5	0.01%
Clostridium botulinum	5	0.01%
unclassified Erysipelotrichia	5	0.01%
Bacteroides heparinolyticus	4	0%
Clostridium ventriculi	4	0%
Enterococcus durans	4	0%
Treponema succinifaciens	4	0%
Sphaerochaeta coccoides	4	0%
Staphylococcus caprae	4	0%
Lachnospira multipara	4	0%
Lactobacillus secaliphilus	4	0%
Eubacterium pyruvativorans	4	0%
Selenomonas sputigena	4	0%
Eubacterium contortum	4	0%
Candidatus Treponema	4	0%
Peptococcus niger	4	0%
Eubacterium infirmum	4	0%
Clostridium neopropionicum	4	0%
Anaerofilum pentosovorans	4	0%
Lactobacillus coleohominis	4	0%
Bacteroides acidifaciens	4	0%
Pseudobutyrivibrio xylanivorans	4	0%
Eubacterium cellulosolvens	4	0%
Oceanirhabdus sediminicola	4	0%
Gottschalkia acidurici	4	0%
Streptococcus thermophilus	4	0%
Bifidobacterium pseudolongum	4	0%
Lactobacillus fermentum	4	0%
Erysipelothrix rhusiopathiae	4	0%
Bacteroides nordii	4	0%
Lactobacillus gallinarum	4	0%
Pediococcus ethanolidurans	4	0%
Asteroleplasma anaerobium	4	0%

Species	Number of reads	Relative abundance
Allisonella histaminiformans	4	0%
Dehalobacterium formicoaceticum	4	0%
Candidatus Heliomonas	4	0%
Staphylococcus capitis	4	0%
Prevotella fusca	4	0%
Geosporobacter ferrireducens	3	0%
Desulfosporosinus orientis	3	0%
Prevotella corporis	3	0%
Desulfotomaculum halophilum	3	0%
Eubacterium ventriosum	3	0%
Defluviitalea raffinosedens	3	0%
Clostridium baratii	3	0%
Bacteroides faecis	3	0%
Mageeibacillus indolicus	3	0%
unclassified Roseburia	3	0%
Clostridium taeniosporum	3	0%
Clostridium paraputrificum	3	0%
Macellibacteroides fermentans	3	0%
Terrisporobacter mayombei	3	0%
Blautia coccoides	3	0%
Lactonifactor longoviformis	3	0%
Bacteroides timonensis	3	0%
Lachnoanaerobaculum saburreum	3	0%
Breznakia pachnodae	3	0%
Sphaerochaeta pleomorpha	3	0%
Bacillus pumilus	3	0%
unclassified Clostridiaceae	3	0%
Casaltella massiliensis	3	0%
Bariatricus massiliensis	3	0%
unclassified Lactobacillaceae	3	0%
Parasporobacterium paucivorans	3	0%
Clostridium indolis	3	0%
Prevotella veroralis	3	0%
Clostridium sufflavum	3	0%
Ruminococcus gauvreauii	2	0%
Parasutterella excrementihominis	2	0%
Adlercreutzia equolifaciens	2	0%
Cytophaga xylanolytica	2	0%
Lachnoanaerobaculum cf.	2	0%
Desulfotomaculum nigrificans	2	0%
Hathewaya limosa	2	0%
Anaerocolumna aminovalerica	2	0%
Garciella nitratireducens	2	0%
Prevotella oulorum	2	0%
Clostridium islandicum	2	0%
Clostridium boliviensis	2	0%
Anaerobiospirillum succiniciproducens	2	0%

Species	Number of reads	Relative abundance
Clostridium aldrichii	2	0%
Clostridioides difficile	2	0%
Clostridium amazonense	2	0%
Slackia exigua	2	0%
Desulfomonas oviles	2	0%
unclassified Wautersiella	2	0%
Streptococcus alactolyticus	2	0%
Caloranaerobacter azorensis	2	0%
Anaerosporobacter mobilis	2	0%
Caproiciproducens galactitolivorans	2	0%
Streptococcus oricebi	2	0%
Treponema bryantii	2	0%
Terrisporobacter petrolearius	2	0%
unclassified Bulleidia	2	0%
Catonella morbi	2	0%
unclassified Oscillospira	2	0%
Clostridium bolteae	2	0%
unclassified Betaproteobacteria	2	0%
Fibrobacter intestinalis	2	0%
Campylobacter hyointestinalis	2	0%
Bacteroides caecicola	2	0%
Mucispirillum schaedleri	2	0%
Clostridium papyrosolvens	2	0%
Howardella ureilytica	2	0%
Streptococcus parasanguinis	2	0%
Bacteroides paurosaccharolyticus	2	0%
Clostridium amygdalinum	2	0%
Eubacterium plexicaudatum	2	0%
Lactobacillus agilis	2	0%
Paraeggerthella hongkongensis	2	0%
Syntrophococcus sucromutans	2	0%
Anaerococcus provenciensis	2	0%
Desulfotomaculum arcticum	2	0%
Campylobacter jejuni	2	0%
Bacteroides clarus	2	0%
Bacteroides gallinaceum	2	0%
Bacteroides oleiciplenus	2	0%
Oxobacter pfennigii	2	0%
Clostridium aminophilum	2	0%
Clostridium straminisolvens	2	0%
unclassified Faecalibacterium	2	0%
Proteinivorax tanatarense	2	0%
Treponema zioleckii	2	0%
Prevotella aff.	2	0%
Streptococcus suis	1	0%
Caldicoprobacter algeriensis	1	0%
Staphylococcus schleiferi	1	0%

Species	Number of reads	Relative abundance
Crassaminicella profunda	1	0%
Clostridium vincentii	1	0%
alpha proteobacterium	1	0%
unclassified Catonella	1	0%
unclassified Collinsella	1	0%
Bacteroides luti	1	0%
Bacillus panaciterrae	1	0%
Peptostreptococcus anaerobius	1	0%
Erythrobacter gangjinensis	1	0%
Solemya pervernicosa	1	0%
Lactobacillus curvatus	1	0%
Clostridium perfringens	1	0%
unclassified Sphingobacterium	1	0%
Pseudoramibacter alactolyticus	1	0%
Bacteroides fragilis	1	0%
Prevotella multiformis	1	0%
Streptococcus mutans	1	0%
Marinilabilia salmonicolor	1	0%
unclassified Methanobrevibacter	1	0%
Caloramator australicus	1	0%
Dethiosulfatibacter aminovorans	1	0%
Clostridium tyrobutyricum	1	0%
Candidatus Methanoplasma	1	0%
Hydrogenoanaerobacterium saccharovorans	1	0%
endosymbiont of	1	0%
Lactobacillus floricola	1	0%
Anaeroplasma abactoclasticum	1	0%
Hungatella effluvii	1	0%
Anaerotruncus colihominis	1	0%
Prevotella oryzae	1	0%
Clostridium aciditolerans	1	0%
Gemella haemolysans	1	0%
Paracoccus lutimaris	1	0%
unclassified Cryptanaerobacter	1	0%
Porphyromonas cangingivalis	1	0%
Clostridiisalibacter paucivorans	1	0%
Synechococcus elongatus	1	0%
Bacteroides vulgatus	1	0%
Streptococcus parasuis	1	0%
Streptococcus salivarius	1	0%
Clostridium carnis	1	0%
Proteocatella sphenisci	1	0%
Clostridium oryzae	1	0%
Bifidobacterium saguini	1	0%
Bacteroides stercorirosoris	1	0%
Geosporobacter subterraneus	1	0%
Tannerella forsythia	1	0%

Species	Number of reads	Relative abundance
Mogibacterium timidum	1	0%
Centipeda periodontii	1	0%
Desnuesiella massiliensis	1	0%
Candidatus Stoquefichus	1	0%
Eubacterium saphenum	1	0%
Olavius environmental	1	0%
Dielma fastidiosa	1	0%
Clostridium septicum	1	0%
Lactobacillus pasteurii	1	0%
Cellulosibacter alkalithermophilus	1	0%
Larkinella bovis	1	0%
Desulfomonile limimaris	1	0%
Atopobium vaginae	1	0%
Clostridium ihumii	1	0%
Proteiniborus ethanoligenes	1	0%
Tyzzerella nexilis	1	0%
Helcococcus kunzii	1	0%
Alkaliphilus metalliredigens	1	0%
unclassified Sutterella	1	0%
Ercella succinigenes	1	0%
Clostridium hungatei	1	0%
Bacteroides zoogleoformans	1	0%
Holdemania massiliensis	1	0%
Peptoclostridium acidaminophilum	1	0%
Paraclostridium bifermentans	1	0%
Streptococcus pharyngis	1	0%
Synergistetes oral	1	0%
Shuttleworthia satelles	1	0%
Helicobacter rodentium	1	0%
Lactobacillus hilgardii	1	0%
Agromyces salentinus	1	0%
Herbivorax saccincola	1	0%
Tepidibacter mesophilus	1	0%
Bacteroidales str.	1	0%
unclassified Prolixibacter	1	0%
Selenomonas lacticifex	1	0%
Fibrobacter succinogenes	1	0%
Desulfovibrio vulgaris	1	0%
Bacillus cereus	1	0%
Rhodospirillum rubrum	1	0%
unclassified Gracilibacter	1	0%
Campylobacter upsaliensis	1	0%
Aeromicrobium ginsengisoli	1	0%
Collinsella massiliensis	1	0%
Alistipes massiliensis	1	0%
Lactobacillus iners	1	0%
unclassified Anaerotruncus	1	0%

Species	Number of reads	Relative abundance
Anaerocolumna jejuensis	1	0%
Clostridium peptidivorans	1	0%
Treponema parvum	1	0%
Clostridium neonatale	1	0%
Bacteroides faecichinchillae	1	0%
Caloramator quimbayensis	1	0%
Caloramator boliviensis	1	0%
Staphylococcus haemolyticus	1	0%
Eisenbergiella massiliensis	1	0%
Pleomorphochaeta multiformis	1	0%
Okadaella gastrococcus	1	0%
Pyramidobacter piscolens	1	0%
Streptococcus infantarius	1	0%
Alkalibacterium putridalgicola	1	0%
Romboutsia lituseburensis	1	0%
unclassified Treponema	1	0%
Staphylococcus hyicus	1	0%
Clostridium viride	1	0%
Bacteroides graminisolvens	1	0%
Dialister propionicifaciens	1	0%
Listeria rocourtiae	1	0%
Kiloniella spongiae	1	0%
Bacteroides salyersiae	1	0%
Propionispira raffinosivorans	1	0%
Propionibacterium namnetense	1	0%
Clostridium oceanicum	1	0%
Streptococcus pasteurianus	1	0%
Acidaminococcus intestini	1	0%
Desulfotomaculum gibsoniae	1	0%
Acholeplasma parvum	1	0%
Sutterella wadsworthensis	1	0%
Rummeliibacillus stabekisii	1	0%
Acetoanaerobium pronyense	1	0%
Atopobium rimae	1	0%
Eubacterium uniforme	1	0%
Slackia piriformis	1	0%
unclassified Erysipelothrix	1	0%
Lactobacillus sakei	1	0%
Helicobacter equorum	1	0%
Lactobacillus intestinalis	1	0%
Corynebacterium tuberculostearicum	1	0%
Prevotella disiens	1	0%
Lactobacillus casei	1	0%
Campylobacter coli	1	0%
Lactobacillus porcinae	1	0%
unclassified Olsenella	1	0%
Thiohalobacter thiocyanaticus	1	0%

Species	Number of reads	Relative abundance
Enterococcus faecalis	1	0%
Paenibacillus hodogayensis	1	0%
Acetatifactor muris	1	0%
Lactobacillus rodentium	1	0%
Eubacterium aggregans	1	0%
Diaminobutyricimonas massiliensis	1	0%
Shigella dysenteriae	1	0%
Staphylococcus hominis	1	0%
Kosakonia sacchari	1	0%
Hathewaya histolytica	1	0%
Clostridium thermosuccinogenes	1	0%
Rhodoglobus vestalii	1	0%
Collinsella intestinalis	1	0%
Anaerostipes caccae	1	0%
Oribacterium parvum	1	0%
Prevotella nigrescens	1	0%
Sedimentibacter acidaminivorans	1	0%
